# 3D genome organization in tissue regeneration involves long-range chromatin loops

**DOI:** 10.1126/sciadv.aea8281

**Published:** 2026-08-07

**Authors:** Palmira Llorens-Giralt, Carlos Camilleri-Robles, Leo Zuber, Jenisha Khadka, Maria Marti-Marimon, Andrea Crespo-Pera, Florenci Serras, Maria Cristina Gambetta, Marc A. Marti-Renom, Montserrat Corominas

**Affiliations:** ^1^ Department of Genetics, Microbiology, and Statistics, School of Biology, Institute of Biomedicine (IBUB), University of Barcelona, Barcelona, Catalonia, Spain.; ^2^ Centre Nacional d’Anàlisi Genòmica (CNAG), Barcelona, Catalonia, Spain.; ^3^ Center for Genomic Regulation (CRG), The Barcelona Institute for Science and Technology (BIST), Barcelona, Catalonia, Spain.; ^4^ Center for Integrative Genomics, University of Lausanne, 1015 Lausanne, Switzerland.; ^5^ ICREA, Barcelona, Catalonia, Spain.

## Abstract

The three-dimensional (3D) organization of the genome plays a fundamental role in gene expression regulation, yet how changes in genome architecture influence transcriptional responses during tissue regeneration remains poorly understood. Here, we used Hi-C to profile 3D chromatin conformation during *Drosophila* wing imaginal disc regeneration. We found that, although compartments and topologically associating domains (TADs) are largely maintained, regeneration is accompanied by reduced compartmentalization and decreased boundary insulation. We identified three long-range chromatin loops with increased contact frequency during regeneration. Targeted deletion of their anchors revealed that these loops are essential for proper disc regeneration but dispensable for normal wing development. Furthermore, disruption of any of these loops resulted in convergent changes in both gene expression and H3K4me1 3D environment, suggesting their coordinated activity during regeneration. These findings provide functional evidence that 3D genome architecture actively contributes to the regenerative process.

## Teaser

Chromosome conformation analysis in *Drosophila* wing discs reveals the requirement of specific meta-loops during regeneration.

## INTRODUCTION

The spatial organization of the genome is increasingly recognized as a crucial regulator of gene expression during cellular differentiation and development (*1*–*3*). High-resolution chromosome conformation capture technologies have revealed that the genome is partitioned into two major compartments, active (A) and inactive (B), which exhibit preferential self-interactions and distinct transcriptional activities (*4*–*6*). A compartments generally correspond to gene-rich, transcriptionally active open chromatin, whereas B compartments represent gene-poor, heterochromatic regions (*5*, *7*). In addition, chromatin folds into functional domains that facilitate regulatory interactions between enhancers and promoters (*6*, *8*). These smaller domains, known as topologically associating domains (TADs), are defined by frequent internal interactions and sharp boundaries that insulate them from the neighboring regions (*6*, *9*). In *Drosophila*, TADs correspond to physical units that correlate well with distinct chromatin states, such as Polycomb-repressed, heterochromatic, or active regions (*10*–*14*). Unlike in mammals, where TAD boundaries are often enriched for CCCTC-binding factor (CTCF) (*5*, *15*), most *Drosophila* TAD borders are associated with active promoters or small domains containing expressed genes (*10*, *16*). Alternatively, nonpromoter TAD boundaries in flies are frequently bound by the architectural protein Centrosomal protein 190 kDa (Cp190) (*17*), although most TADs show little apparent change in the absence of Cp190 (*18*). Recent studies have also identified long-range chromatin loops, high-frequency interactions spanning hundreds of kilobases to a few megabases, whose anchors often overlap with TAD boundaries and are likely stabilized by architectural proteins (*19*). These loops contribute to transcriptional regulation by enabling the precise spatiotemporal control of neuronal gene expression in the central nervous system (*20*, *21*) and supporting the cross-regulation of paralogous genes during embryogenesis (*22*, *23*). Other long-range loops are established by Polycomb complexes and can facilitate either activation or repression of developmental genes (*24*–*26*).

Evidence from different species and injury contexts suggests that regenerative potential is closely linked to chromatin state dynamics and enhancer activation (*27*, *28*). Following tissue damage, neighboring cells integrate signals from the wound, triggering transcriptional reprogramming and widespread chromatin remodeling to enable effective tissue repair (*29*, *30*). However, whether this reprogramming involves higher-order chromatin reorganization, such as changes in TADs or chromatin loops, remains unclear. *Drosophila* imaginal discs, epithelial tissues capable of compensatory regeneration, offer a powerful model to address this question. Their cellular plasticity, combined with an extensive genetic toolkit, has enabled the identification of key signaling and epigenetic factors involved in tissue repair (*31*–*33*). The early stages of wing disc regeneration are characterized by a reduction in repressive histone marks, increased chromatin accessibility and transcriptional activation (*29*). These regenerative gene programs are driven by damage-responsive regulatory elements, enhancers that are preferentially or specifically activated by injury (*29*, *34*). Despite these advances, little is known about the contribution of three-dimensional (3D) chromatin architecture in regulating injury-induced gene expression. Understanding how 3D genome topology changes in response to damage could reveal new principles of gene regulation during tissue regeneration.

Here, we investigate the 3D regulatory landscape of wing disc regeneration by generating Hi-C interaction maps in control and genetically ablated discs. We find that while global genome organization is largely preserved following injury, chromatin compartmentalization is notably weakened. In addition, we identify three long-range chromatin loops that exhibit significantly increased interaction frequency during regeneration. Deletion of the intergenic anchors of each loop impairs regeneration but does not affect normal development, demonstrating their specific requirement for tissue repair. Moreover, disruption of any of these loops results in similar transcriptomic changes and predicted convergent alterations in the 3D H3K4me1 landscape, suggesting that they contribute to a shared chromatin regulatory environment that may influence gene expression during regeneration. We further show that these loops are anchored by Cp190, which may be recruited to loop anchors by the insulator-binding factor Ibf2. Together, our findings identify a previously unknown role for genome architecture in tissue repair, revealing an additional regulatory layer of gene expression during regeneration.

## RESULTS

### Global changes in 3D chromatin architecture during regeneration

**Fig. 1.**
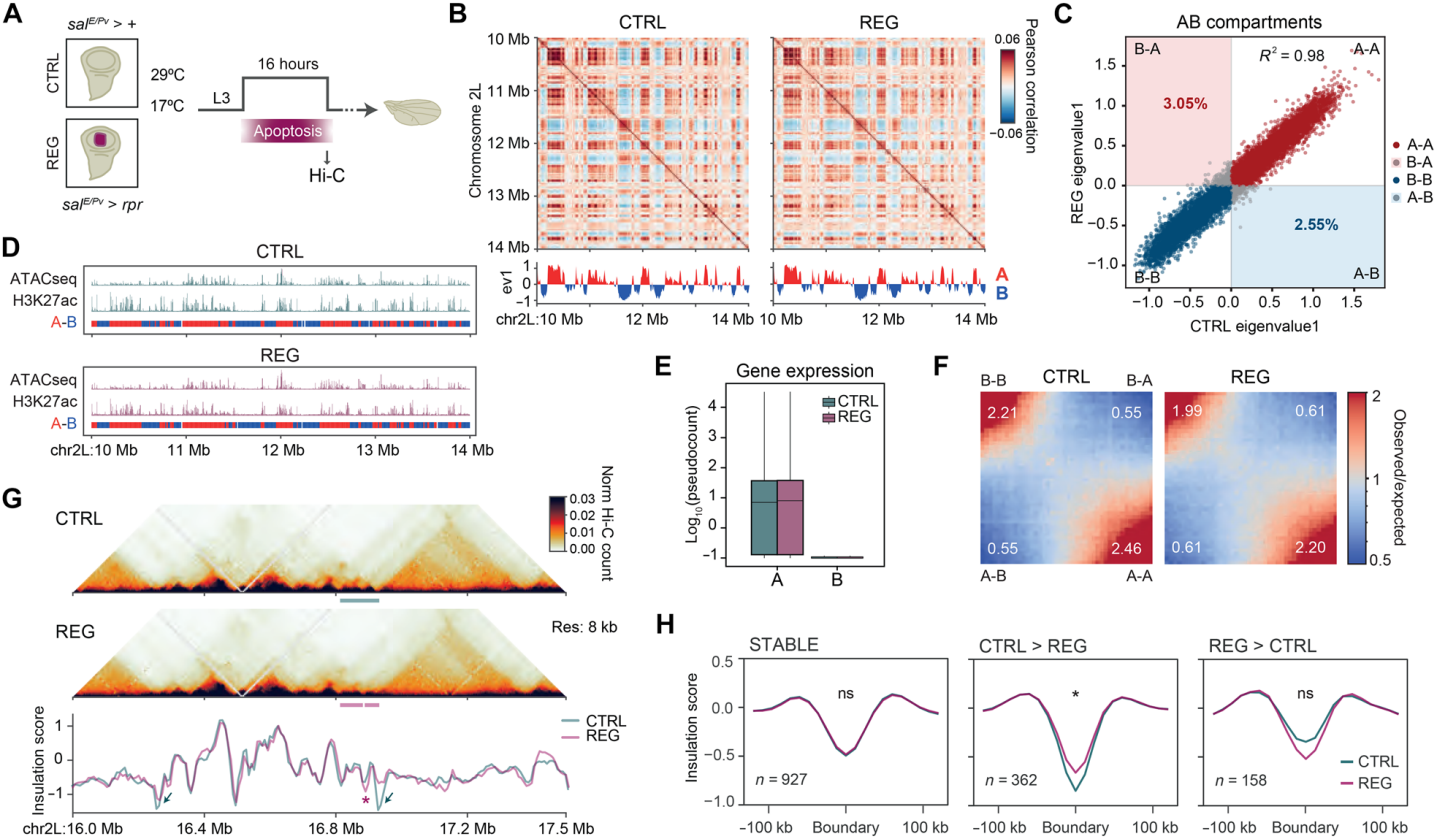
A/B compartment strength and boundary insulation is mildly reduced during regeneration. (**A**) Experimental design. Flies were raised at 17°C until 192 hours AEL (corresponding to the third- instar larval stage, L3) then shifted to 29°C for 16 hours to induce cell death via *rpr* expression in the *sal^E/Pv^* domain of the wing pouch (purple region). Wing discs were dissected immediately after treatment and processed for Hi-C. Controls without *rpr* expression were treated in parallel. (**B**) A/B compartment analysis showing Pearson correlation matrices and eigenvectors for chromosome 2L in control (CTRL) and regeneration (REG). (**C**) Scatter plot comparing pairwise compartment eigenvectors between CTRL and REG. (**D**) Genome browser tracks displaying ATAC-seq and H3K27ac ChIP-seq signals intensities (*29*), alongside compartment classifications: A (red) and B (blue). (**E**) Gene expression levels of genes located in A or B compartments in CTRL and REG (*37*). (**F**) Saddle plots showing genome-wide compartmentalization strength. A-A and B-B interactions are stronger in CTRL than in REG. (**G**) Hi-C contact maps with insulation scores for CTRL and REG illustrating two boundaries with significantly increased insulation in CTRL (arrows) and a de novo TAD boundary emerging in REG (asterisk). TAD length in the region of interest is shown below each Hi-C map. (**H**) Pairwise comparison of insulation scores across boundaries categorized as stable, increased in CTRL (CTRL > REG), or increased in REG (REG > CTRL). ns, not significant.

To study whether chromatin changes during wing disc regeneration are associated with a reorganization of genome topology, we performed Hi-C to map the 3D genome architecture of wing discs after genetic ablation. Cell death was induced for 16 hours by expressing the proapoptotic gene *reaper* (*rpr*) in the *spalt* (*sal^E/Pv^*) domain of the wing pouch in third-instar larvae (*35*, *36*). Wing discs were collected immediately after *rpr* expression was switched off, and two biological replicates were processed for in situ Hi-C (Fig. 1A). The high correlation between replicates allowed us to merge them into a single dataset per condition (fig. S1A).

To analyze changes in 3D chromatin architecture, we first identified A/B compartments in control (CTRL) and regenerating (REG) discs at a 10-kb resolution. These compartments reflect broad chromatin states, with A generally associated with active, accessible regions and B with inactive, condensed chromatin (*4*, *8*). Compartment analysis revealed a strong correlation between the two conditions, with 56 and 57% of compartments classified as A, and 44 and 43% classified as B in CTRL and REG samples, respectively (Fig. 1B and fig. S1B). Compartmental switches during regeneration were infrequent, with 2.55% of the genome changing from A to B, and 3.05% switching from B to A (Fig. 1C). These changes are three to five times smaller (fig. S2, A to C) than those observed when comparing CTRL or REG samples with central nervous system tissue at a similar larval stage (*20*), or during embryo development in *Drosophila* (*23*). To further characterize the A/B compartments, we integrated our previously published Assay for Transposase-Accessible Chromatin with high-throughput sequencing (ATAC-seq), H3K27ac chromatin immunoprecipitation sequencing (ChIP-seq), and RNA sequencing (RNA-seq) datasets from REG wing discs (*29*, *37*). As expected, A compartments were enriched for accessible chromatin, H3K27ac, and actively expressed genes, whereas B compartments predominantly contained inactive chromatin and nonexpressed genes (Fig. 1, D and E). In addition, B compartments exhibited greater differences between conditions than A compartments (fig. S1C). Notably, we observed weakened compartment strength following damage (Fig. 1F and fig. S1D).

To assess changes at the level of domains, we calculated insulation scores and identified TAD boundaries at 8-kb resolution. Boundary insulation profiles were globally similar between CTRL and REG discs (Fig. 1G), with 64% of TAD boundaries remaining stable and showing no statistical differences in insulation score distributions (paired *t* test, *P* > 0.05 for 927 boundaries; Fig. 1H). Among boundaries exhibiting changes, most insulation differences were modest, with a larger fraction showing significantly decreased insulation during regeneration (paired *t* test, *P* < 0.05 for 362 boundaries; Fig. 1H), whereas fewer boundaries displayed increased insulation (paired *t* test, *P* > 0.05 for 158 boundaries; Fig. 1H). Boundaries with increased insulation were evenly distributed between quantitative gains and newly formed (de novo) TAD boundaries, which typically showed small increases in insulation (Fig. 1G and fig. S1E). In contrast, most boundaries with decreased insulation reflected quantitative reductions that were more pronounced and overall significant (Fig. 1G). De novo and preexisting boundaries had similar genomic distributions, with ∼75% located at gene promoters (fig. S1G). As with compartments, insulation score changes between CTRL and REG were modest compared to those observed when comparing CTRL or REG with central nervous system tissue at a similar larval stage (fig. S2, D and E) (*20*) or during embryonic development in flies (fig. S2F) (*23*). Together, these results suggest that genome organization is largely preserved during regeneration, accompanied by a mild reduction of A/B compartmentalization, weakened insulation at certain TAD borders, and the emergence of a small subset of new, relatively weak boundaries.

### Enrichment of specific long-range chromatin loops during regeneration

**Fig. 2.**
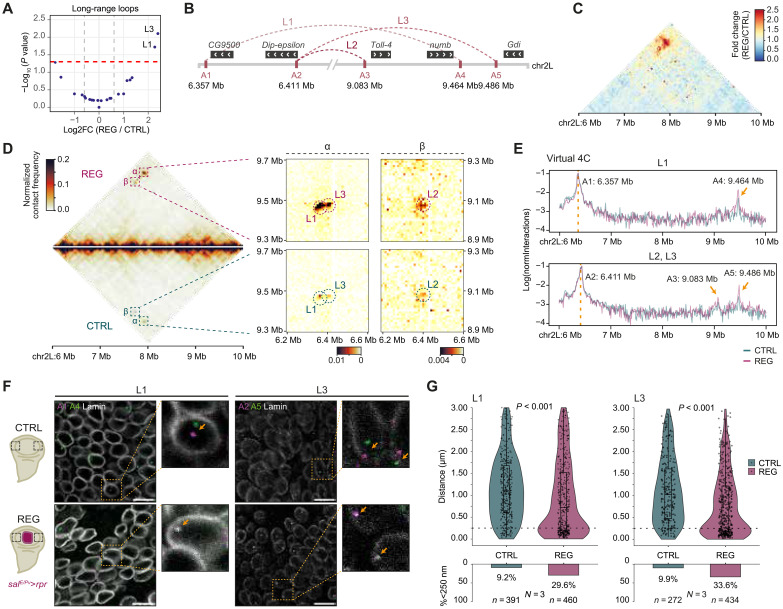
Specific long-range chromatin loops are enriched during regeneration. (**A**) Differential analysis of meta-loop strength in CTRL versus REG wing discs. Labeled loops were significantly stronger (fold change > 1.5 and *P* < 0.05) during regeneration. (**B**) Schematic representation of loops L1–L3, their corresponding anchors A1–A5, and the nearest gene associated with each anchor. (**C**) Hi-C fold change map (REG/CTRL) showing differential contacts across chr2L: 6 to 10 Mb. (**D**) Left: Mirror plot of normalized Hi-C contact maps for REG (top) and CTRL (bottom), with two regeneration-enriched regions highlighted. Right: Zoom-in views of the alpha (α) and beta (β) regions from the REG (top) and CTRL (bottom) Hi-C maps, showing loops L1, L2, and L3. (**E**) Virtual 4C plots generated from Hi-C data using A1 as bait to visualize loop L1 (top) and A2 to detect loops L2 and L3 (bottom) under REG and CTRL conditions. (**F**) Schematics of CTRL and REG wing discs showing the regions imaged for DNA-FISH indicated by squares. Right: Representative zoomed-in DNA-FISH images labeled with probes targeting A1 (magenta) and A4 (green) for loop L1 (left), and A2 (magenta) and A5 (green) for loop L3 (right). Lamin staining (white) marks the nuclear lamina. Scale bars: 5 μm. (**G**) Quantification of DNA-FISH distances (in micrometers) for L1 (left) and L3 (right) anchors. Violin plots show the distribution of measured distances, and the percentage of nuclei with distances <250 nm is indicated below. *n* denotes the number of nuclei analyzed; *N* indicates the number of biological replicates.

Chromatin loops appear as interaction hotspots in high-resolution Hi-C maps, reflecting frequent physical contacts between distant genomic loci (*38*). To identify regeneration-specific loops, we applied an automated loop-calling algorithm (*20*) in both CTRL and REG Hi-C datasets. Following manual validation, we identified 27 curated long-range loops (>1 Mb) in the wing disc (table S1). Among these, only two loops showed significant enrichment in regeneration: L1, spanning loop anchors A1 to A4 (chr2L:6,357,000–9,464,000), and L3, between A2 and A5 (chr2L:6,411,000–9,486,000) (Fig. 2A). Anchor A2 was also involved in a third loop, L2, connecting A2 and A3 (chr2L:6,411,000–9,083,000), but did not exhibit significant enrichment. These three loops were previously characterized as meta-loops, high-frequency interactions within meta-domains, which are defined as TAD pairs separated by megabases that preferentially interact in the central nervous system, and a subset of which also exists in wing discs (*20*). To precisely map the meta-loop anchors, we used previously published ATAC-seq data in CTRL and REG wing discs (*29*), assigning each anchor to accessible chromatin regions (fig. S3A). With the exception of A2, which was located at the *DIP-epsilon* promoter, all anchors mapped to intergenic regions: A1 near the 3′ end of *CG9500*, A3 upstream of *Toll-4*, A4 near the 3′ end of *numb*, and A5 downstream of *Gdi* (Fig. 2B and fig. S3A). The anchor regions for loops L1–L3 were consistently more accessible in REG compared to CTRL, showing higher mean accessibility than anchors from other loops (fig. S3B), suggesting that increased chromatin accessibility may contribute to the formation or stabilization of these specific meta-loops during regeneration. Visual inspection of the REG/CTRL fold change Hi-C matrix revealed an interaction hotspot spanning around 3 Mb on chromosome 2L (Fig. 2C), and specific inspection of the individual Hi-C maps showed consistent enrichment of loops L1 and L3 during regeneration (Fig. 2D). Virtual 4C analysis further supported these findings, showing a strong enrichment of L1 and L3 during regeneration, but not of L2 (Fig. 2E).

Given the regeneration-specific enrichment of L1 and L3, we next assessed their spatial proximity and cell-to-cell variability by measuring physical distances between meta-loop anchors in CTRL and REG wing discs using DNA fluorescence in situ hybridization (DNA-FISH). We designed probes targeting the two anchors of each loop and quantified the distance between A1 and A4 (L1), and between A2 and A5 (L3) within individual nuclei, considering distances below 250 nm as indicative of a physical interaction (*39*). DNA-FISH images were acquired from hinge cells adjacent to the ablated pouch (Fig. 2F), which constitute the regeneration-competent population that proliferates and reprograms to restore the tissue (*40*). This analysis revealed a significant increase in the proportion of nuclei with physically close anchors during REG compared to CTRL: 29.6 versus 9.2% for L1 and 33.6 versus 9.9% for L3 (Fig. 2, F and G). These results are consistent with our Hi-C data, indicating that although these loops are present in a minority of cells in CTRL discs, their interaction frequency significantly increases during regeneration. This suggests that meta-loop formation is specifically enriched in wound-adjacent, regeneration-responsive cells and could reflect a transient chromatin reorganization that peaks during the early regeneration phase, when reprogramming is actively occurring (*36*, *40*). As a control, we performed DNA-FISH of a previously characterized meta-loop connecting the *nolo* promoter with an intergenic region located ∼1.5 Mb away on chromosome 2L, detected in both wing discs and the central nervous system of third-instar larvae (*20*). This loop was also observed in our CTRL and REG Hi-C maps (fig. S3C) and was identified by the meta-loop calling algorithm (table S1). Quantitative analysis of the DNA-FISH results confirmed the reliability of our approach in detecting physical chromatin interactions (fig. S3D).

To explore whether particular DNA motifs contribute to the preferential formation of L1–L3 during regeneration, we performed a de novo motif discovery on anchor sequences using STREME, followed by a comparison with known motifs using TOMTOM and FIMO from the MEME suite (*41*). This approach identified five candidate motifs, three of which could be annotated to known transcription factor (TF) binding sites: Dorsal, Giant, and CG11617. All five motifs were significantly enriched in L1–L3 anchors compared to those of L4–L28 (table S2), suggesting that regeneration-associated meta-loops may be stabilized by specific sequence features, potentially reflecting the recruitment of DNA binding factors to their anchors.

To determine whether the increased interaction frequency of L1 and L3 during regeneration is associated with transcriptional changes, we analyzed the expression of genes located at loop anchors or within the corresponding TADs using transcriptomic data already available from REG wing discs (*37*). For L1, we found that *CG9500* was not expressed, while *numb* was highly expressed in both CTRL and REG wing discs, with no significant change (fig. S3E). At the L3 anchors, *DIP-epsilon* showed a trend toward up-regulation, while *Gdi* remained highly expressed under both conditions, and *Toll4*, located near A3, was not expressed under either condition. Among genes within the meta-domains, only *Tig* was significantly up-regulated, while the others showed no significant differential expression (fig. S3E). *Tig* (*Tiggrin*) encodes an extracellular-matrix protein and is required for muscle attachment and plasmatocyte maturation, but no roles in epithelial damage response have been reported.

### Regeneration-enriched meta-loops are required for wing disc regeneration

**Fig. 3.**
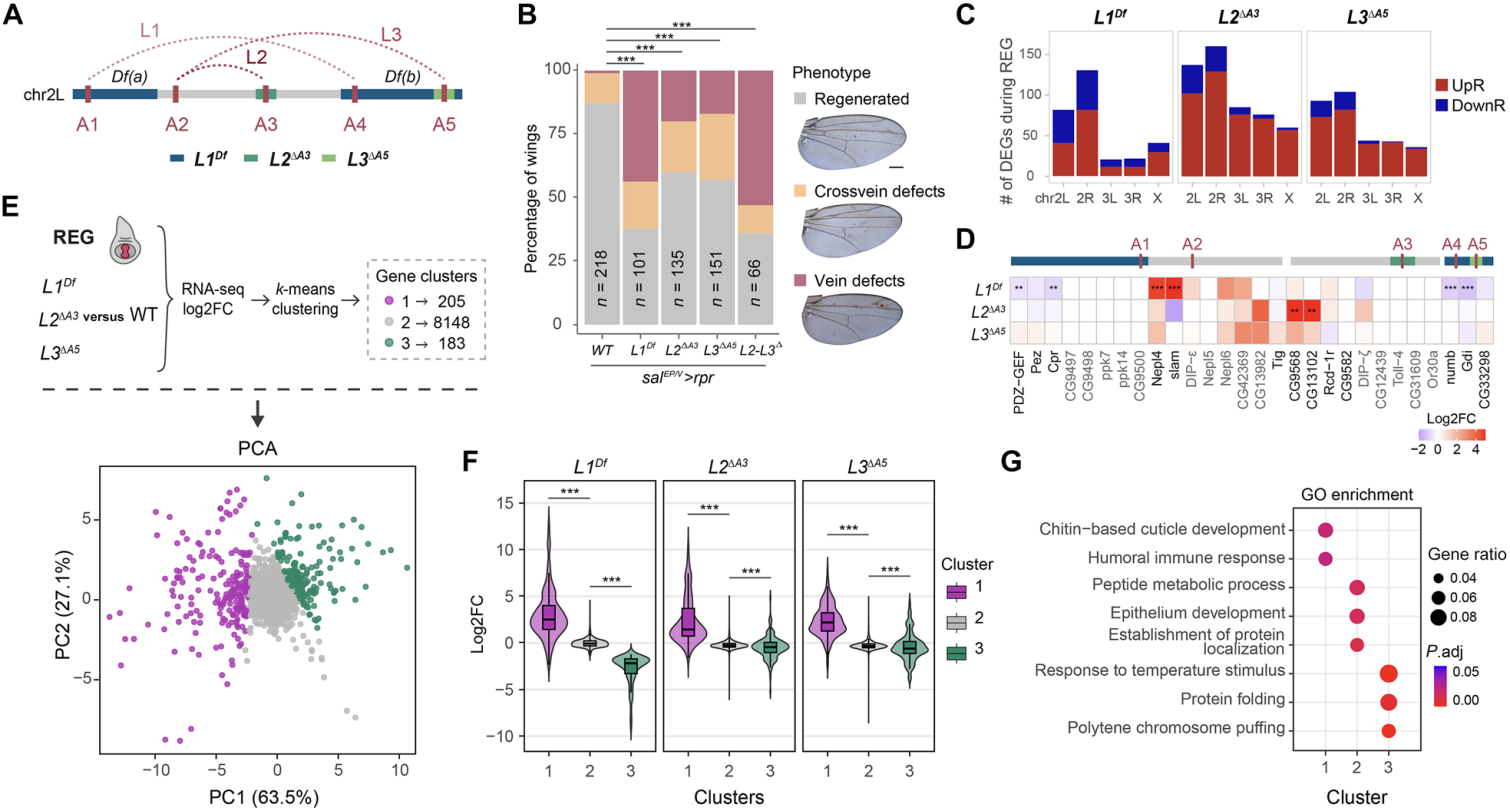
Meta-loops L1, L2, and L3 are required for regeneration. (**A**) Schematic representation of *L1^Df^*, *L2*^Δ*A3*^, and *L3*^Δ*A5*^ genotypes. *L1^Df^* carries chromosomal deficiencies *Df(a)* and *Df(b)* in trans-heterozygosity, while *L2*^Δ*A3*^ and *L3*^Δ*A5*^ are generated through precise CRISPR-mediated deletion of anchors A3 and A5, respectively. (**B**) Percentage of adult wing phenotypes across genotypes: fully regenerated wings (gray), crossvein defects (yellow), and vein defects (rose). Representative images of each phenotype are shown to the right. (**C**) Total number of DEGs per genotype during regeneration, grouped by chromosome. Up-regulated (UpR) genes are shown in red, and down-regulated (DownR) genes are shown in blue. (**D**) Heatmap representing gene expression changes in *L1^Df^*, *L2*^Δ*A3*^, and *L3*^Δ*A5*^ transcriptomes. Genes located within meta-TADs containing A1–A5 are represented. Names of nonexpressed genes (<1 TPM in all samples) are labeled in light gray. (**E**) Top: Schematic overview of the gene clustering strategy used on *L1^Df^*, *L2*^Δ*A3*^, and *L3*^Δ*A5*^ transcriptomes. The number of genes per cluster is indicated. Bottom: PCA based on log 2–transformed gene expression changes, with genes colored by the *k*-means cluster assignment: cluster 1 (purple), cluster 2 (gray), and cluster 3 (green). (**F**) Violin plots showing log 2–transformed gene expression changes for each gene cluster, grouped by transcriptome. (**G**) GO term enrichment for each gene cluster. Statistical significance indicated in (B), (D), and (F): ***P < 0.001; **P < 0.01.

To investigate the functional relevance of L1 and L3 meta-loops in wing discs, we disrupted their anchor sites using two different approaches. For L1, we used available chromosomal deficiencies: *Df(2L)BSC186* (chr2L:6,253,005–6,363,074), which encompasses anchor A1, and *Df(2L)Exel6022* (chr2L:9,447,643–9,560,489), which spans anchor A4 (L1) and A5 (L3), hereafter referred to as *Df(a)* and *Df(b)*, respectively (Fig. 3A). As both deficiencies are homozygous lethal, we analyzed their effects in heterozygotes. To assess the impact of L1 disruption, we used trans-heterozygous flies (*L1^Df^*) carrying one copy of each deficiency. We reasoned that the heterozygous loss of both anchor sites would disrupt intrachromosomal meta-loop formation while preserving the expression of hemizygous genes. Of note, none of the genes overlapping either deficiency showed differential expression in wild-type REG wing discs (fig. S4A). *L1^Df^* flies developed normally, with no observable defects in adult wing size or morphology (fig. S4B). Similarly, flies heterozygous for either *Df(a)* or *Df(b)* alone [*L1^Df(a)^* and *L1^Df(b)^*] also exhibited no visible phenotypic abnormalities (fig. S4B).

To specifically disrupt the L3 meta-loop, we used CRISPR-Cas9 to precisely delete the ATAC-seq peak corresponding to the intergenic A5 anchor (Fig. 3A). We avoided deleting A2, as it overlaps with the promoter of *DIP-epsilon*. Since L3 shares the A2 anchor with the L2 meta-loop, we also generated CRISPR knockout (KO) flies for the intergenic A3 anchor, which is specific to L2. To minimize potential off-target effects, we generated two independent CRISPR deletion alleles for each anchor and crossed the corresponding alleles to obtain trans-heterozygous flies (hereafter referred to as *L2*^Δ*A3*^ and *L3*^Δ*A5*^). Because each homologous chromosome carries an independent deletion of the same anchor, these animals are effectively homozygous for the deletion. Both CRISPR lines were viable and displayed normal adult wing size and morphology (fig. S4B).

We next assessed whether the disruption of these meta-loops impairs tissue repair after damage. We induced apoptosis in the wing pouch by genetically activating *rpr*, as previously described (Fig. 1A). Following ablation, *L1^Df^*, *L2*^Δ*A3*^, and *L3*^Δ*A5*^ mutant flies exhibited a significant reduction in regenerative capacity. *L1^Df^* flies showed the lowest proportion of regenerated wings (38%), followed by *L3*^Δ*A5*^ (57%) and *L2*^Δ*A3*^ (60%), compared to regenerating controls (87%) (Fig. 3B). All three mutants also exhibited a significantly higher proportion of severe phenotypes relative to controls, characterized by major disruptions in vein patterning such as complete vein loss or multiple defects in veins and crossveins. Flies carrying the *L1^Df(b)^* single deficiency also showed increased regeneration defects, although a larger fraction (23.6%) presented milder phenotypes, such as missing or ectopic crossveins (fig. S4C). Although the deficiencies span multiple genes, and thus we cannot exclude the possibility that impaired regeneration in *L1^Df^* results from haploinsufficiency of one or more genes, these findings strongly suggest that regeneration-enriched meta-loops are required for proper regeneration, but dispensable for normal development. Moreover, to determine whether the simultaneous disruption of both L2 and L3 meta-loops enhances the regeneration defects, we generated double KO flies carrying deletions of both the A3 and A5 anchors. Using the strategy described above, we produced two independent double-deletion alleles and crossed them to obtain trans-heterozygous flies in which each homologous chromosome carries an independent deletion of both anchors (hereafter referred to as *L2-3*^Δ*A3-5*^ double KO). These double KO flies were viable and displayed normal adult wing size and morphology, further indicating that these regions are not essential for wing development under homeostatic conditions. However, following *rpr*-induced apoptosis, *L2-3*^Δ*A3-5*^ double KO flies exhibited a stronger impairment in regeneration compared to single mutants, with a further reduction in the proportion of fully regenerated wings and an increased frequency of severe wing defects (Fig. 3B). These results suggest that L2 and L3 meta-loops act cooperatively to regulate tissue regeneration.

To characterize the molecular changes associated with meta-loop disruption, we performed RNA-seq on regenerating wing discs from flies harboring single deficiencies [*L1^Df(a)^* and *L1^Df(b)^*], trans-heterozygous flies carrying both deficiencies (*L1^Df^*), and CRISPR-mediated KOs (*L2*^Δ*A3*^ and *L3*^Δ*A5*^), using wild-type regenerating wing discs as controls. The spatial distribution of samples revealed that single-deficiency flies clustered together, whereas *L1^Df^*, *L2*^Δ*A3*^, and *L3*^Δ*A5*^ samples formed distinct clusters (fig. S4, D and E). Consistent with the phenotypic observations, *L1^Df^* flies showed the highest number of differentially expressed genes (DEGs) compared to controls (264 genes), followed by *L1^Df(b)^* (194 genes) and *L1^Df(a)^* (87 genes) (fig. S4F). In the CRISPR mutants, we identified 518 DEGs in *L2*^Δ*A3*^ and 320 in *L3*^Δ*A5*^ (fig. S4G). Across all mutants, most DEGs were up-regulated and preferentially located on chromosome 2, likely reflecting local structural perturbation since the disrupted meta-loops are anchored within chromosome 2L (Fig. 3C). Furthermore, most DEGs in *L3*^Δ*A5*^ were also found in *L2*^Δ*A3*^ (fig. S4G), suggesting that L2 and L3 may contribute to common regulatory functions, likely through repression of gene expression. This is further supported by the strong correlation in gene expression changes between the two CRISPR mutants (Pearson correlation = 0.612).

Visual chromosomal inspection of *L1^Df^*, *L2*^Δ*A3*^, and *L3*^Δ*A5*^ revealed a scattered distribution of DEGs along chromosome 2L (fig. S4H). Specifically, genes *CG9500*, *DIP-epsilon*, and *Toll-4*, associated with anchors A1, A2, and A3, respectively, were not expressed under any condition [<1 transcript per million (TPM)] (Fig. 3D). In contrast, genes near A4 and A5 (*numb* and *Gdi*) were down-regulated in *L1^Df^*, but showed no significant changes in expression in *L2*^Δ*A3*^ or *L3*^Δ*A5*^. These findings suggest that regeneration-enriched meta-loops contribute to broad domain-wide regulation of gene expression, likely through structural and/or insulating functions, rather than by directly controlling the expression of nearby anchor-associated genes.

To determine whether the global transcriptional changes induced by meta-loop disruption are shared across the *L1^Df^*, *L2*^Δ*A3*^, and *L3*^Δ*A5*^ mutants, we integrated transcriptomic data from regenerating wing discs of all three conditions (Fig. 3E). Unsupervised clustering of genes based on their expression changes revealed three distinct clusters: Cluster 1 genes were consistently up-regulated, Cluster 2 genes showed minimal expression changes, and Cluster 3 genes were predominantly down-regulated across loop-deficient samples (Fig. 3, E and F, and table S3). We next examined how these clusters behave during wild-type regeneration (*37*). Most genes differentially expressed in regeneration belonged to Cluster 2, showing minimal changes in the mutants (fig. S4I). Notably, a modest inverse relationship was observed: Genes down-regulated during regeneration tended to map to Cluster 1 (7.1%, up-regulated in mutants) rather than Cluster 3 (4.3%, down-regulated in mutants), whereas genes up-regulated during regeneration were more frequently found in Cluster 3 (6.1%) than Cluster 1 (2.2%) (fig. S4I). These results suggest that meta-loop disruption does not broadly alter the regenerative expression program.

We further characterized the genomic context of these clusters relative to 3D architectural features. Genes from Clusters 1 and 3 overlapped with significantly fewer TAD boundaries compared to Cluster 2 (fig. S4J). However, among the genes that did overlap boundaries, Clusters 1 and 3 were enriched at boundaries with altered insulation, whereas Cluster 2 genes preferentially overlapped stable boundaries (fig. S4, K and L). Moreover, although most genes in all clusters resided in active A compartments as expected, Clusters 1 and 3 were more frequently located in inactive B compartments compared to Cluster 2 genes (fig. S4M). Together, these data indicate a positional bias of Clusters 1 and 3 relative to Cluster 2 with respect to both TAD boundaries and A/B compartmentalization.

Functional enrichment analysis showed that Cluster 1 was significantly enriched for genes involved in chitin-based cuticle development and the humoral immune response (Fig. 3G). Since these genes are likely repressed by the regeneration-enriched meta-loops, we speculate that the repression of genes involved in cuticle development may help maintain the epithelial tissue plasticity before pupariation, while the regulation of immune response genes could prevent inflammation-derived damage during regeneration. Cluster 2 was enriched for genes linked to developmental and metabolic functions that may not be directly involved in regeneration, and Cluster 3 genes were enriched for Gene Ontology (GO) terms related to responses to stimuli, suggesting a possible role of these meta-loops in regulating stress-related gene expression. Together, the transcriptomic similarities observed across L1, L2, and L3 disruptions suggest that these regeneration-enriched chromatin loops function in a coordinated manner, primarily by repressing gene expression.

### In silico deletion of individual meta-loop anchors leads to convergent changes in the 3D H3K4me1 environment

**Fig. 4.**
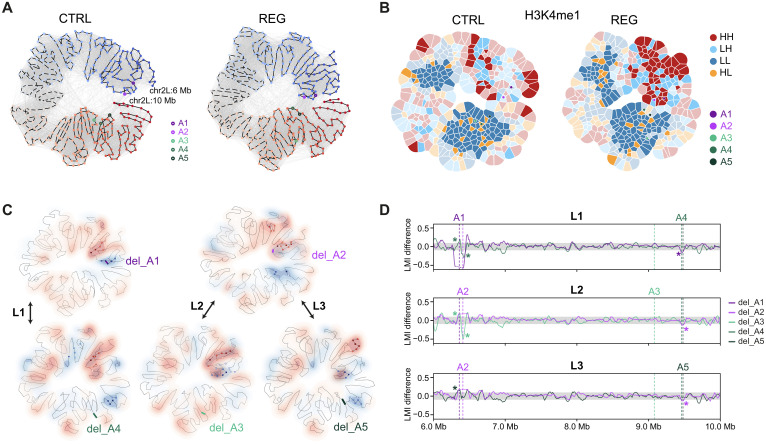
Predicted deletion of meta-loop anchors leads to convergent changes in the 3D H3K4me1 landscape. (**A**) 2D Kamada-Kawai graph layout of CTRL and REG wing disc samples, representing a 4-Mb region containing A1–A5 (chr2L: 6 to 10 Mb). Each node corresponds to a 10-kb genomic bin. (**B**) Gaudí plots projecting H3K4me1 signals (*29*) and spatial classification of each bin based on the LMI. Bins are color-coded to indicate quadrant classification and statistical significance (solid colors, *P* < 0.05). Bins are classified as: high-high (HH), enriched in signal and surrounded by similarly enriched bins; low-low (LL), depleted in signal and surrounded by depleted bins; high-low (HL), enriched in signal-poor neighborhoods; and low-high (LH), depleted within signal-rich neighborhoods. (**C**) Predicted Kamada-Kawai graph layout of REG discs upon deletion of ten 10-kb bins centered on each anchor region. Colors indicate LMI differentials compared to wild-type REG discs: Red regions show higher H3K4me1 signals in deleted discs, while blue regions show lower H3K4me1 environments. Dots represent the bins showing the 95th percentile LMI differential scores. (**D**) Representation of the predicted LMI differential scores for each position in the linear genome. Colored lines indicate smoothed LMI changes for each deletion. Asterisks indicate changes in the H3K4me1 environment at the anchor opposite the deleted anchor.

To better understand how disruption of L1–L3 meta-loops influences gene expression, we examined whether these structural alterations could affect the 3D regulatory environment. We focused on a 4-Mb region of chromosome 2L spanning anchor regions A1–A5 (chr2L: 6 to 10 Mb). Using Hi-C data, we first generated a spatial layout of this region in both CTRL and REG wing discs using the Kamada-Kawai algorithm (*42*). Consistent with previous observations (Fig. 2, F and G), anchors A1–A4 (L1) and A2–A5 (L3) were closer in REG, while anchors A2–A3 (L2) appeared in closer proximity in CTRL discs (Fig. 4A).

Next, we integrated Hi-C and available ATAC-seq and ChIP-seq datasets from REG wing discs (*29*) to interrogate the 3D chromatin environment of this region. We applied METALoci (*43*), a computational method pipeline to assess spatial autocorrelation of epigenetic signals within a defined genomic region by integrating Hi-C contact data and epigenomic marks (H3K4me1, H3K27ac, or ATAC). Using the Kamada-Kawai layout algorithm, METALoci constructs a spatial graph in which each node represents a 10-kb genomic bin and edges reflect physical chromatin interactions, allowing the mapping and statistical analysis of epigenetic signal clustering via Local Moran’s *I* (LMI) algorithm (*44*). METALoci predicted notable differences in the H3K4me1 landscape in REG discs compared to CTRL (Fig. 4B), whereas minimal changes were observed for the levels of H3K27ac and chromatin accessibility (fig. S5, A and B). In particular, the 3D region containing anchors A1 and A3 showed enriched H3K4me1 signal during regeneration, suggesting a shift toward a more active chromatin environment, while the A3–A5 region remained in a low H3K4me1 state.

Subsequently, we explored how in silico deletion of individual meta-loop anchor sites would reshape the H3K4me1 regulatory landscape. To this end, we used the METALoci’s scan module, which performs in silico deletions of 100-kb regions centered at the meta-loop anchors by removing nodes and their associated interactions from the layout. For each modified configuration, the layout is recomputed and the LMI recalculated, enabling detection of shifts in epigenetic autocorrelation patterns relative to the wild-type topology. These predictions revealed convergent changes in the 3D H3K4me1 environment regardless of which meta-loop anchor region was deleted, with Pearson’s correlation coefficients (PCCs) of over 0.92 between any two deletions (Fig. 4, C and D, and fig. S5C). Specifically, deletion of a given meta-loop anchor consistently altered the H3K4me1 environment of the opposite anchor. For instance, deletion of A1 or A2 resulted in lower H3K4me1 signal at the A4–A5 domain, while deletion of A3–A5 significantly altered H3K4me1 levels at the A1–A2 3D region (Fig. 4, C and D). Although deleting A3 altered the A1–A2 predicted environment, the H3K4me1 status of the A3 region was not affected when other anchors were deleted. Last, we analyzed whether these predicted changes in the H3K4me1 environment correlated with gene expression changes observed upon L1, L2, and L3 disruption. While no clear correlation was found between predicted H3K4me1 changes and overall gene expression levels (fig. S5D), up-regulated genes tended to reside within 3D regions showing higher H3K4me1 signal compared to down-regulated genes (fig. S5E). Together, the convergent changes observed in both gene expression and the 3D H3K4me1 environment upon meta-loop disruption suggest that L1, L2, and L3 act coordinately during regeneration.

### Architectural proteins mediating regeneration-associated meta-loops

**Fig. 5.**
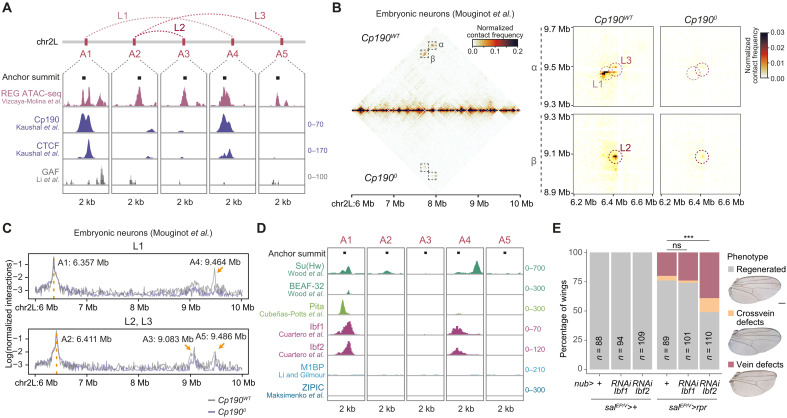
Cp190 mediates the formation of regeneration-associated meta-loops. (**A**) Genome browser tracks of published ATAC-seq data from REG wing discs (*29*) and ChIP-seq profiles of indicated insulator proteins (*46*, *47*) across a 2-kb window containing anchors A1–A5. The anchor summit represents the 150-bp region assigned to each anchor region based on the mean signal from ATAC-seq data. Above, schematic overview of meta-loops L1–L3 and their corresponding anchors A1–A5. The ATAC-seq profile is scaled to view for each anchor. (**B**) Micro-C contact map analysis in wild-type and Cp190-deficient embryonic neurons (maternal depletion and KO, *Cp190*^*0*^) (*21*). Left: Mirror plot of normalized Micro-C contact maps for chromosome 2L (6 to 10 Mb). Right: Zoom-in views of the α and β regions containing loops L1 and L3, and L2, respectively. (**C**) Virtual 4C plots with A1 as bait to visualize loop L1 (top) and A2 to detect loops L2 and L3 (bottom). Loop anchor coordinates are indicated. (**D**) Genome browser tracks of ChIP-seq profiles of indicated tethering proteins (*48*–*52*) across a 2-kb window containing anchors A1–A5. (**E**) Proportion of adult wing phenotypes across genotypes (*nub* > +, *nub* > *Ibf1^RNAi^*, and *nub* > *Ibf2^RNAi^*), during development (*sal^E/Pv^* > +) and regeneration (*sal^E/Pv^* > *rpr*), categorized as fully regenerated wings (gray), crossvein defects (yellow), and vein defects (rose). Representative images of each phenotype are shown to the right. Statistical significance is indicated: ***P < 0.001.

Meta-loops are thought to arise from interactions between structural elements, rather than from enhancer-promoter looping, and be stabilized by dedicated transcription factors and architectural proteins (*20*, *21*). To identify the factors involved in L1, L2, and L3 meta-loop formation, we examined ChIP-seq profiles for architectural proteins known to establish long-range regulatory interactions (*20*, *21*, *45*, *46*). Specifically, we examined Cp190 and CTCF ChIP-seq data from larval neurons (*47*), where these loops are also present, as well as GAGA-associated factor (GAF) data from wing discs (*46*). This analysis revealed binding of Cp190 and CTCF at L1 anchor regions A1 and A4, whereas the anchors for L2 and L3 (A2, A3, and A5) were not bound by any of these factors (Fig. 5A). To determine whether Cp190 or CTCF are required for the formation of L1, we analyzed publicly available Hi-C data from embryos lacking maternal and zygotic Cp190 (*Cp190^0^*) (*21*), and CTCF mutant (*CTCF^0^*) larval neurons (*47*). Consistent with Cp190 occupancy at its anchors, the L1 meta-loop was lost in *Cp190^0^* embryos (Fig. 5, B and C). Notably, L2 and L3 were also strongly disrupted in these mutants (Fig. 5, B and C), suggesting that they either directly depend on Cp190 or require the formation of L1 to establish themselves. In contrast, contact frequencies across these three meta-loops were preserved in *CTCF^0^* larvae (fig. S6A). Similarly, L1, L2, and L3 architecture remained intact in wing discs from *GAF* mutant flies (fig. S6, B and C) (*46*). *Cp190* was not differentially expressed during wing disc regeneration, based on RNA-seq data (fig. S6D) (*37*), and other Cp190-dependent loops previously identified (*21*) were not enriched in the REG Hi-C matrix (Fig. 2A). This suggests that the increased interaction frequencies of L1, L2, and L3 during regeneration may be driven by a specific, context-dependent mechanism, rather than a general increase in Cp190 activity.

Because Cp190 lacks a DNA binding domain, we sought to identify the tethering element responsible for recruiting Cp190 to L1 anchors. We analyzed binding profiles for several known Cp190-recruiting proteins, including suppressor of hairy wing [Su(Hw)], boundary element–associated factor of 32 kDa (BEAF-32) (*48*), and Pita (*49*) in Kc167 cells, and insulator-binding factors 1 and 2 (Ibf1/Ibf2) (*50*), motif 1 binding protein (M1BP) (*51*), and zinc-finger protein interacting with CP190 (ZIPIC) (*52*) in S2 cells, all of which have been previously shown to mediate looping by recruiting Cp190 to chromatin (*48*–*50*, *52*). Similar to Cp190, Ibf1 and Ibf2 showed binding at A1 and A4 (Fig. 5D), but not at A2, A3, or A5. No binding of BEAF-32, M1BP, or ZIPIC was observed at any of the L1–L3 anchors, and Su(Hw) binding was detected only at A1 and adjacent to A4. This suggests that Ibf1 and Ibf2 may be responsible for recruiting Cp190 to the L1 anchors. To test their functional relevance during regeneration, we assessed their expression and requirement during regeneration. Neither gene was differentially expressed during regeneration (fig. S6D). However, using RNA interference (RNAi)–mediated knockdown of *Ibf1* or *Ibf2* specifically in the *nubbin* region of the wing disc while simultaneously inducing apoptosis in the wing pouch by genetically activating *rpr* as previously described (*53*), we found that loss of *Ibf2* significantly impaired wing regeneration, whereas *Ibf1* knockdown had no effect (Fig. 5E). No phenotypic effects were observed for either gene in the absence of cell death induction (Fig. 5E). These results identify Ibf2 as a key regulator specifically during regeneration, possibly through its role in Cp190 recruitment to regeneration-associated L1 meta-loop. Together, these findings indicate that Cp190 is required for the formation of the L1 meta-loop, potentially recruited to its anchors by the insulator-binding factor Ibf2.

## DISCUSSION

In this study, we investigated how genome architecture and long-range chromatin loops contribute to tissue regeneration. Following damage to the wing disc, we observed weakened A/B compartmentalization and reduced insulation at certain TAD boundaries, likely facilitating increased interactions between neighboring TADs. Despite these localized changes, global genome organization remained largely stable, with no major evidence of substantial compartment switching. The observed changes were overall limited compared to those detected when contrasting our samples with other tissues or developmental stages (*20*, *23*). This is consistent with previous findings in both human and *Drosophila* cells, where TADs and compartments remain mostly unaltered after stress, except for a general reduction in TAD boundary strength (*54*–*56*).

Furthermore, we identified three long-range chromatin loops, two of which showed increased contact frequency after damage, and demonstrated their requirement for successful wing disc regeneration. Although disruption of these three damage-induced loops results in regeneration defects, these phenotypes are relatively mild when compared to those caused by mutations in core regeneration-specific genes (*29*, *40*, *57*). This suggests that meta-loops may exert subtle, context-dependent regulatory effects, modulating transcription in a more fine-tuned manner rather than driving strong, tissue-wide gene activation.

Notably, *L1^Df^*, *L2*^Δ*A3*^, and *L3*^Δ*A5*^ mutant flies displayed no obvious developmental abnormalities or defects in adult wing morphology in the absence of injury, despite the presence of these loops during embryogenesis (*23*) and in larval neurons (*20*), which suggests a regeneration-specific function. To explain this context-specific requirement, we propose two models: (i) Meta-loops mediate regulatory interactions that remain latent during development but are specifically activated in response to damage, or (ii) meta-loops play redundant roles during development, where regulatory networks are robust to minor perturbations, but become essential during regeneration, when precise transcriptional control is critical and there is less tolerance for alterations. Supporting the first model, it has been previously described that sequences lacking intrinsic enhancer activity, termed distal tethering elements (DTEs), may form focal contacts with promoters prior to transcriptional activation (*58*). These loops facilitate enhancer-promoter interactions and prime genes for rapid activation, thereby enabling temporal precision in gene expression. While deletion of DTEs disrupts these loops and delays gene activation, the overall transcriptional output remains largely unaffected (*58*). Similarly, Polycomb complexes can form long-range contacts that support regulatory interactions between enhancers and genes, keeping them in a silenced but poised state (*26*). Conversely, recent studies suggest that meta-loops do not directly establish enhancer-promoter interactions but instead facilitate regulatory cross-talk between distant TADs by enabling the sharing of regulatory elements and transcription factors, as their intergenic anchors generally lack intrinsic enhancer activity (*20*, *21*). In line with this, transcriptomic analysis of REG wing discs from *L1^Df^*, *L2*^Δ*A3*^, and *L3*^Δ*A5*^ mutant flies revealed changes in gene expression extending beyond the anchor-associated genes. Most DEGs were up-regulated upon loop disruption, particularly in *L2*^Δ*A3*^ and *L3*^Δ*A5*^ flies, suggesting that these meta-loops may facilitate transcriptional repression during homeostasis. This potential repressive activity may be particularly relevant during regeneration, where it could help maintain tissue plasticity prior to pupariation and prevent inflammation-induced damage. None of the anchor-associated genes were differentially expressed in the *L2*^Δ*A3*^ and *L3*^Δ*A5*^ mutants, nor are they known to function in wing disc regeneration. Moreover, our in silico analyses indicate that the three meta-loops act in a coordinated manner to establish a 3D chromatin regulatory landscape, with meta-domains flanking anchors A1 and A2 residing in an active chromatin state, and those near A3–A5 residing in a repressive state. Predicted deletion of any meta-loop anchor perturbs this 3D environment, particularly around the opposite anchor site, but also more broadly across chromosome 2L. Together, these results support a model in which regeneration-enriched meta-loops act primarily as architectural elements that maintain proper chromatin organization and contribute to broad transcriptional regulation, facilitating rather than driving the regenerative response. Alternatively, these meta-loop disruptions could also involve subtle regulatory changes that are not captured by bulk transcriptomic assays. Therefore, high-resolution approaches such as single-molecule RNA-FISH or quantitative single-cell live imaging (*20*–*22*) may be necessary to fully elucidate how meta-loops influence gene expression during regeneration. This coordinated activity, supported by similarities in both transcriptomic data and 3D simulation predictions across the three mutants, may help explain why L2 disruption leads to phenotypic anomalies similar to those caused by L1 and L3, despite L2 not being enriched in wild-type regenerating wing discs.

Consistent with its known role in stabilizing long-range chromatin contacts (*21*), Cp190 was found to be required for the formation of all three regeneration-associated meta-loops. Considering that Cp190 binding is detected at L1 but not at L2 and L3, we suggest a hierarchical assembly model in which the Cp190-mediated L1 loop is established first and facilitates the subsequent formation of L2 and L3. This model aligns with previous findings showing that intergenic-intergenic loops often precede intergenic-promoter interactions during meta-loop assembly within the same domain (*20*, *21*). Although we have not directly addressed which factors localize Cp190 at L1 anchor sites, available Hi-C data from CTCF, BEAF-32, and GAF mutants suggest that these factors are likely not required for Cp190 recruitment (*20*, *46*, *59*). Instead, we hypothesize that Ibf2 may bind to the L1 anchors and promote Cp190-dependent loop formation, subsequently enabling the assembly of L2 and L3 meta-loops.

In conclusion, our study provides evidence that changes in 3D genome architecture are associated with wing disc regeneration and supports a functional contribution of long-range chromatin loops to successful regeneration. However, several limitations should be considered. Although Hi-C analyses in mutant backgrounds could, in principle, offer additional structural information, we anticipate that the expected changes would be highly localized and therefore difficult to resolve in population-averaged maps, providing limited mechanistic insight for our study. Similarly, high-resolution imaging approaches in genetically perturbed flies across regenerative time points will be important to directly assess how anchor deletions or Ibf2 depletion affect spatial chromatin organization during regeneration. These experiments would also determine whether such structural changes are transient or restored at later stages of tissue repair. In addition, our METALoci analysis should be interpreted as a conceptual framework rather than a definitive map of mutant chromatin states, since the predicted H3K4me1 shifts are subtle and not validated experimentally. Future studies across different regenerative contexts and species will be key to determining whether reduced chromatin compartmentalization is a conserved feature of injury responses, how changes in genome structure drive transcriptional reprogramming toward regenerative gene expression programs, and the specific contribution of architectural proteins in coordinating tissue repair. Furthermore, advances in single-cell resolution Hi-C technologies will help unravel the dynamics of genome organization across different cell types, including at the single-cell level, within the context of tissue repair.

## MATERIALS AND METHODS

### *Drosophila* strains

The following *Drosophila melanogaster* strains were used: *UAS-rpr* (*60*), *LexO-rpr* and *sal^E/Pv^-LHG* (*53*), *sal^E/Pv^-Gal4* (*35*), and *nub-Gal4* (*61*). The following were acquired from the Bloomington Drosophila Stock Center: *w^1118^* (RRID:BDSC_5905), *tub-Gal80^TS^* (RRID:BDSC_7017), *Df(2L)BSC186* (RRID:BDSC_9614), and *Df(2L)Exel6022* (RRID:BDSC_7506). The following were acquired from the Vienna Drosophila Resource Center: *UAS-Ibf1-RNAi* (RRID:VDRC_104542) and *UAS-Ibf2-RNAi* (RRID:VDRC_42121).

### Genetic ablation and dual Gal4/LexA transactivation system

Cell death was induced as previously described (*35*, *36*). For Hi-C and DNA-FISH experiments, expression of the proapoptotic gene *reaper* (*rpr*) was driven using *sal^E/Pv^-Gal4* in combination with the thermosensitive repressor *tub-Gal80^TS^*. For deficiencies, CRISPR, and RNAi experiments, genetic ablation was induced using *sal^E/Pv^-LHG*, *LexO-rpr* and *tub-Gal80^TS^* strains. RNAi expression was induced using a second transgene, *nub-Gal4*, also repressed by tub-*Gal80^TS^*. For wing regeneration assays, embryos were maintained at 17°C until 180 hours after egg laying (AEL) to prevent *rpr* expression. They were then shifted to 29°C for 11 hours to induce cell death and then back to 17°C until adulthood to assess wing regeneration. This timing consistently yields >75% fully regenerated wings in control animals, providing a robust baseline for phenotypic comparisons. To collect wing discs, embryos were kept at 17°C until 192 hours AEL and then moved to 29°C for 16 hours to induce *rpr* expression. Wing discs were immediately collected afterward for processing. This longer ablation period aligns with the onset of larval wandering behavior prior to pupariation, ensuring collection at a consistent and physiologically relevant time point. Control samples without *rpr* expression were always treated in parallel.

### Test for adult wing phenotypes

Adult female flies were fixed in a 1:2 glycerol:ethanol solution for 24 hours. After fixation, wings were dissected in water and subsequently washed with ethanol. The wings were then mounted in 6:5 lactic acid:ethanol and imaged under a Leica DMLB optical microscope. Aberrant wings were classified based on vein defects: Strong aberrations were defined as missing veins or multiple defects, while mild aberrations were characterized by missing or extra crossveins. The frequency of regenerated versus nonregenerated wings for each genotype was statistically compared using Fisher’s exact test. Bonferroni correction was applied when multiple comparisons were tested.

### CRISPR-Cas9–mediated intergenic anchor deletions

Loop anchors were precisely deleted as previously described (*20*), by CRISPR-Cas9–mediated genome editing using up to four small guide RNAs (sgRNAs) for each region chosen for deletion: 537 base pairs (bp; dm6 coordinates chr2L:9,082,818–9,083,354) for *L2*^Δ*A3*^ and 779 bp (dm6 coordinates chr2L:9,486,161–9,486,939) for *L3*^Δ*A5*^. sgRNAs were cloned using primers listed in table S4. For each anchor, flies harboring independently isolated CRISPR KO alleles were first crossed to flies carrying either *LexO-rpr* or *sal^E/Pv^-LHG*, *tub-Gal80^TS^*. The resulting offspring was crossed to generate trans-heterozygous flies carrying an independent deletion on each homologous chromosome, effectively homozygous for the anchor loss. Wild-type regenerating controls were generated in parallel using the same parental stocks carrying the regeneration system. Consequently, control and mutant animals shared the same X and third chromosome backgrounds and differed only at the second chromosome carrying the CRISPR anchor deletions. These flies were then assessed for wing phenotypes.

### In situ Hi-C library preparation and sequencing

In situ Hi-C experiments from wing discs were performed in duplicate as previously described (*25*, *62*). Briefly, ∼200 wing discs for each replicate were quickly dissected (<1 hour) from wandering larvae at room temperature (RT) in Schneider’s insect medium before being directly processed. Wing discs were washed twice in phosphate-buffered saline (PBS) and resuspended in A1 buffer [60 mM KCl, 15 mM NaCl, 4 mM MgCl_2_, 15 mM Hepes, 0.5% Triton X-100, 0.5 mM dithiothreitol, 1× protease inhibitor cocktail (Sigma-Aldrich), and H_2_O]. Then, formaldehyde was added to reach a 1.8% final concentration and transferred on a rotating wheel for 10 min at RT. Formaldehyde was quenched with 1.25 M glycine, and samples were washed twice with ice-cold A1 buffer. Supernatant was discarded, and fixed wing discs were air-dried and then snapped frozen in liquid nitrogen to process all samples in parallel. For all samples, wing discs were thawed on ice and resuspended in ice-cold A1 buffer. The samples were homogenized on ice using a tight Tenbroeck, centrifuged, and resuspended in 0.5% SDS for 10 min at 62°C to permeabilize nuclei. SDS was quenched by adding Triton X-100 and incubated for 15 min at 37°C with rotation. All enzymatic steps were performed within intact nuclei to minimize contributions from fragmented or apoptotic cells. Nuclei integrity was checked after each major step to ensure that nuclei remained intact and structurally preserved. Next, samples were resuspended in lysis buffer for a 30-min incubation on ice, and nuclei were subjected to Mbo I treatment overnight at 37°C in NEB2.1 buffer. The next day, restriction sites were end-repaired and biotinylated using Klenow [New England BioLabs (NEB), catalog no. M0210] and biotin-14-dATP (Life Technologies, catalog no. 19524-016) before being re-ligated using T4 DNA ligase (NEB, catalog no. M0202) overnight at 16°C. The next day, samples were treated with ribonuclease (RNAse) A for 15 min at 37°C and then incubated with proteinase K and reverse cross-linked for 6 hours at 65°C. Subsequently, DNA was purified using AMPure XP beads (Beckman Coulter, catalog no. A63880) and then sheared to obtain fragments of an average size of 300 to 400 bp using the Bioruptor Pico (Diagenode; 1 μg of DNA in 100 μl; six cycles; 20” ON, 60” OFF). For library preparation, biotinylated DNA was pulled down by adding an equal volume of Dynabeads MyOne Streptavidin T1 beads (Life Technologies, catalog no. 65602). Biotin was removed from unligated ends, and pulled-down DNA fragments were end-repaired and A-tailed with NEBNext A-tailing module (E6053L). Illumina adaptors were ligated, and libraries were amplified by eight cycles of polymerase chain reaction (PCR) using NEBNext High-Fidelity 2× PCR Master Mix (catalog no. M0541S). DNA was size-selected using AMPure XP beads (Beckman Coulter, catalog no. A63880) at a ratio of beads to library of 0.9:1 to isolate 300- to 800-bp fragments. Last, the quality of the libraries was visualized with the Agilent Bioanalyzer High Sensitivity DNA Assay (Agilent Technologies, Savage, DE) and checked by low sequencing depth on a NextSeq 500 prior to higher sequencing depth on NovaSeq 6000 platform at the Center for Genomic Regulation (CRG) sequencing facility in Barcelona, Spain. A minimum of 500 million paired-end 150-bp-long reads were obtained per sample.

### DNA fluorescence in situ hybridization

DNA-FISH was performed as previously described with minor modifications (*20*). Probes were prepared by nick translation. First, 5-kb PCRs from genomic DNA were cloned into a plasmid. Next, 2 μg of miniprep plasmid DNA was nick-translated (Abbott Molecular 07J100-001) following the manufacturer’s protocol in the presence of 10 mM aminoallyl-dUTP-XX-ATTO-488 (Jena Bioscience NU-803-XX-488-S) or aminoallyl-dUTP-ATTO-550 (Jena Bioscience NU-803-550-S) at 15°C for 5 hours and stored at −20°C. Prior to DNA-FISH, both probes per two-color DNA-FISH experiment were precipitated separately in the presence of salmon sperm DNA and resuspended in 100% formamide. Samples were prepared by dissecting L3 larval cuticles in ice-cold PBS 1× and subsequently fixing in 4% formaldehyde for 30 min at RT and washing 3× with PBT (PBS with 0.1% Triton X-100), 1× with PBT:MeOH (1:1), and 1× with MeOH, and then stored in 100% MeOH at −20°C. Samples were rehydrated in 2×SSCT (30 mM sodium citrate dihydrate, pH 7.4, 300 mM NaCl, and 0.1% Tween 20), treated with RNase A for 30 min at RT, washed in 2×SSCT, and permeabilized with PBS–0.5% Triton for 15 min at RT. Next, samples were washed in 2×SSCT, incubated in freshly prepared 0.2 N HCl, washed in 2×SCCT, and incubated for 2 hours in 50% formamide/2×SSCT at 37°C. DNA-FISH probes in 80% formamide and larval cuticles in 50% formamide/2×SSCT were predenatured by incubating for 10 min at 80°C and immediately cooled on ice. Then, probes were mixed with the larval cuticles in 25 μl total of 2×SSC, 10% (w/v) dextran sulfate, 0.1% Tween 20, and 50% formamide, heated to 80°C for 10 min, and then incubated at 37°C overnight in the dark. Larval cuticles were then washed in 50% formamide in 2×SSCT (2 × 30 min at 37°C), 20% formamide in 2×SSCT (20 min at 37°C), 2×SSCT (2 × 5 min at 37°C), and 2×SCTT (5 min at RT). Samples were then incubated in PBS, 0.1% Tween 20, 1× Western blocking reagent (Sigma-Aldrich, 1921673) (Blocking Buffer with Tween-20, BBT) for 30 min at RT and immunostained with anti-lamin (mouse monoclonal clone ADL67.10, Developmental Studies Hybridoma Bank) diluted 1:10 in BBT overnight at 4°C. The next day, samples were washed in BBT and then incubated for 1 hour at RT with Alexa 647 anti-mouse immunoglobulin G (Thermo Fisher Scientific, A21235) diluted 1:200 in BBT. Samples were washed in PBS with 0.1% Tween 20 (3 × 10 min) and finally mounted with 4′,6-diamidino-2-phenylindole to stain DNA. Images were acquired on a Leica DMi8 inverted fluorescence microscope using a 63× oil objective and visualized with Fiji software v2.1.0/1.53c. Three independent biological replicates were analyzed for each condition. To quantify the distance between anchors, nuclei were 3D segmented using the lamin channel using Cellpose cyto3 model (*63*). Image preprocessing and detection of individual FISH spots were performed using IMARIS as previously described (*19*). Nuclei with only one spot for each channel were selected, and the shortest distance between the center of each DNA-FISH spot (each anchor) was 3D measured, considering <250 nm as interacting loci (*39*). Statistical analysis was performed in R using the Shapiro-Wilk test to assess normality, followed by a Mann-Whitney *U* test to compare between CTRL and REG.

### Hi-C data analysis

Hi-C data production, from raw FASTQ files through to interaction matrices, was conducted using the TADbit pipeline (*64*). The workflow began with quality control checks on the raw FASTQ data. Sequencing reads were then mapped using the GEM mapper (*65*) to the *D. melanogaster* reference genome (dm6). Mapping was done using an iterative alignment strategy (*66*). Following mapping, reads were subjected to a series of filters to eliminate artifacts, such as nonspecific ligations, sequencing errors, and other experimental anomalies. Specifically, TADbit’s default filtering applied nine distinct criteria: self-circles, dangling ends, errors, extra dangling ends, overrepresented fragments, overly short or long fragments, duplicates, and random breaks. Next, the data were normalized using the ICE (Iterative Correction and Eigenvector decomposition) balancing method (*66*). The resulting valid read pairs were binned at multiple resolutions (2 kb, 4 kb, 5 kb, 8 kb, 10 kb, 20 kb, 25 kb, 50 kb, 100 kb, 500 kb, and 1 Mb), with normalization biases and decay corrections incorporated to construct interaction matrices. Table S5 summarizes the number of valid read pairs, and the number of reads that was filtered out per replicate. Replicate datasets were compared and merged using TADbit’s merging function, which incorporates the HiCRep similarity score (*67*).

### A/B compartment analysis

A/B compartments were identified as previously proposed (*4*) in 10-kb normalized Hi-C matrices using cooltools (*68*) and cooler (*69*). Each individual chromosome arm was analyzed separately, and bins around centromeres were excluded (*47*) (chr2L:1–22,170,000; chr2R:5,650,000–25,286,936; chr3L:1–22,900,000; chr3L: 4,200,000–32,080,000; and chrX:1–23,542,271). The first eigenvector of the correlation matrix was obtained by principal components analysis (PCA) of the observed-over-expected matrix. Bins with positive eigenvector values were assigned to A and bins with negative values to B compartments. The saddle plots were generated using cooltools and show the interaction frequency of intra- or intercompartments, representing the compartment strength or level of compartmentalization for each condition.

### Insulation analysis

Insulation scores were calculated in the 8-kb normalized, merged matrices using FAN-C (*70*) (parameters: window size 80 kb, threshold 0.4). Bins around centromeres were excluded (*47*). Bedtools intersect function was used to assess which boundaries were present or absent in CTRL and REG.

### Comparison with published Hi-C datasets

FASTQ files from datasets GSM6614564, GSM6614565, and GSM6614566 were downloaded from National Center for Biotechnology Information Gene Expression Omnibus (*20*). These datasets correspond to replicates of Hi-C experiment in L3 larval state for the central neuronal system of *Drosophila*. These experiments were parsed as above described with an identical protocol as for the CTRL and REG samples. A/B compartment and insulation scores were compared, and differences in TAD boundaries were assessed using a paired *t* test analysis for boundaries that were deemed stable (less than 0.1 insulation score differences), increasing (<0.1 differences), or decreasing (>0.1 differences). Similarly, to assess the differences of A/B compartment and insulation score observed during development, the ArrayExpress datasets E-MTAB-12070 and E-MTAB-13267 corresponding to 2 to 4 and 16 to 18 hours AEL (*23*) were downloaded and parsed as above indicated.

### Meta-loop analysis

Meta-loops were called by applying the loop calling algorithm from the meta-loops-22 repository (available at https://github.com/gambettalab/meta-loops-2022/) (*20*). As the input, we used the balanced Hi-C interaction matrix for REG and CTRL conditions in .mcool format and set the following parameters: resolution = 2000, score-tresh = 35, and clustering-distance = 3. Then, we intersected the called meta-loops under each condition to get a single matrix containing the coordinates of all identified meta-loops. After manual curation, we filtered out meta-loops shorter than 1 Mb. To precisely map the anchor site summit, we used the ATAC-seq data from CTRL and REG wing discs (*29*). First, we computed the nucleosome-free region (NFR) signal by averaging the signal from the CTRL and REG replicates. Then, we expanded the 2-kb bin containing the anchor site by ±1 kb and divided the resulting 4 kb into 150-bp sliding windows every 25 bp. We computed the mean NFR signal for each window and assigned as the anchor summit the coordinates of the window showing the highest mean signal. The final list of identified meta-loops with each anchor summit is shown in table S1.

For differential loop strength analysis, we used a nonparametric, bootstrapping-based approach. We generated 10,000 randomly shifted loops preserving the same loop sizes of the original identified meta-loops. For each condition, the strength of both real and random loops was calculated as the total number of Hi-C contacts (raw counts) of the 14-kb window centered on the 2-kb bin containing the summit of both meta-loop anchors. For each loop, we computed the log 2–transformed fold change (log2FC) of contact strength between conditions (REG/CTRL), using a pseudocount of 1. We used the log2FC distribution of random loops to construct the empirical null distribution. Then, we calculated the empirical two-sided *P* values for each real loop by comparing its absolute log2FC to the null distribution.

### De novo motif analysis at chromatin loop anchors

To assess motif enrichment at chromatin loop anchors, we performed a de novo motif discovery analysis using the MEME suite (v5.5.8). Anchor coordinates were expanded to 500 bp centered on the midpoint of each anchor summit. From the set of 28 identified long-range chromatin loops (table S1), five anchors corresponding to meta-loops L1–L3 were defined as the positive set, while the remaining 50 anchors (loops L4–L28) were used as background. BED files were generated for each group and converted to FASTA format using the dm6 reference genome.

Motif discovery was carried out with STREME, using the positive set as foreground and the background anchors as negative sequences, with the following parameters: motif width 6 to 15 bp, Markov order 2, and 0.1 discovery threshold. Identified motifs were annotated with TOMTOM against the JASPAR CORE Insect database (v2020), retaining the best match per query motif based on E-value score. Motif occurrences across all anchor sequences were mapped with FIMO using a threshold of 1 × 10^−4^. We then classified motif hits into L1–L3 anchors versus L4–L28. For each motif, we quantified the number of anchors with at least one occurrence and tested for enrichment in L1–L3 anchors using Fisher’s exact test. *P* values were corrected for multiple testing using the Benjamini-Hochberg false discovery rate. Results were summarized in table S2, reporting each motif and its consensus sequence, annotation status, distribution across anchor groups, and statistical significance. The mean expression values of the TF associated with each motif in TPMs are also reported for CTRL and REG wing discs (*37*).

### RNA-seq library preparation and sequencing

Embryos of the appropriate genotypes were maintained at 17°C until 192 hours AEL. They were then shifted to 29°C for 16 hours to induce *rpr* expression. Third-instar larvae (L3) from the following genotypes were selected for the RNA-seq experiments shown in Fig. 3 and fig. S4: *w^−^; +; sal^E/Pv^-LHG:tub-Gal80^TS^/lexO-rpr* (*WT*), *w^−^; Df(2L)BSC186/+*; *sal^E/Pv^-LHG:tub-Gal80^TS^/lexO-rpr* [*L1^Df(a)^*], *w^−^; Df(2L)Exel6022/+*; *sal^E/Pv^-LHG:tub-Gal80^TS^/lexO-rpr* [*L1^Df(b)^*], *w^−^; Df(2L)BSC186/Df(2L)Exel6022*; *sal^E/Pv^-LHG:tub-Gal80^TS^/lexO-rpr* (*L1^Df^*), *w^−^; L2*^Δ*A3*^*; sal^E/Pv^-LHG:tub-Gal80^TS^/lexO-rpr* (*L2*^Δ*A3*^), and *w^−^; L3*^Δ*A5*^; *sal^E/Pv^-LHG:tub-Gal80^TS^/lexO-rpr* (*L3*^Δ*A5*^). Forty wing discs per sample were dissected in cold Schneider’s medium. The Quick-RNA Microprep Kit (Zymo Research) was used following the manufacturer’s instructions, including a 15-min deoxyribonuclease incubation, to isolate the RNA. The purity and concentration of the resulting RNA were assessed using NanoDrop (Thermo Fisher Scientific) and Bioanalyzer (Agilent Technologies).

For the library preparation, 500 ng of total RNA was used for reverse transcription. Ribosomal RNA was depleted by poly-A selection. All libraries were sequenced on an Illumina NextSeq2000 sequencer, using 50-bp paired-end reads. Library preparation and sequencing were performed at the Genomics Unit of the CRG. Samples were sequenced in two independent sequencing batches: one for the conditions *WT*, *L1^Df(a)^*, *L1^Df(b)^*, and *L1^Df^*, and one for the conditions *WT*, *L2*^Δ*A3*^, and *L3*^Δ*A5*^.

### RNA-seq data analysis

Reads were aligned to the *D. melanogaster* dm6 genome using STAR v2.7.10b (*71*), with up to four mismatches per alignment, using the FlyBase genome annotation version r6.62. Reads mapping to more than 10 loci were discarded. Gene expression was quantified in the number of counts using the featureCounts function from the Rsubread package v2.22.1 (*72*) in R. Genes whose sum of counts in all replicates was lower than 10 were discarded.

To account for batch effects between the two sequencing runs, the rlog-transformed expression matrix was corrected using the ComBat function from the sva package v3.48.0 (*73*). The correction model was first estimated using control samples (wild-type, WT), which were present in both batches, and subsequently applied to all conditions.

For dimensionality reduction, we applied both PCA and *t*-distributed stochastic neighbor embedding (*t*-SNE). PCA was performed on the ComBat-corrected rlog expression matrix to assess global variance structure across replicates. Since PCA did not fully resolve replicate separation after batch correction, we additionally used *t*-SNE (*74*) implemented in the Rtsne package v0.17. Prior to *t*-SNE, the top 2000 variable genes were selected to reduce noise. The algorithm was run with a perplexity of 6, a maximum of 10,000 iterations, and a fixed random seed to ensure reproducibility. To evaluate the effectiveness of batch correction, silhouette scores were computed based on Euclidean distances in the *t*-SNE space, yielding a mean value of 0.013, consistent with minimal residual batch clustering.

Differential expression analysis (DEA) was performed using DESeq2 package v1.40.2 (*75*) in R. We did two independent DEAs: one for the L1 deficiencies and one for the CRISPR-Cas9 deletions. Only genes with at least 1 TPM in at least one sample were selected for each DEA. A generalized linear model was fitted with the design formula ∼ condition, where condition is a factor corresponding to each genotype. The control genotype (*w^−^; +; sal^E/Pv^-LHG:tub-Gal80^TS^/lexO-rpr*) was used as the reference level, and differential expression was assessed by comparing each of the other genotypes to the reference. The Benjamini-Hochberg method was used to adjust *P* values for multiple testing. All genes with an absolute fold change >2 and an adjusted *P* < 0.05 were considered differentially expressed.

For *k*-means clustering of genes, previously calculated log 2–transformed expression fold change values for the following conditions were scaled and used as input: *L1^Df^*, *L2*^Δ*A3*^, and *L3*^Δ*A5*^. Genes expressed <10 counts in at least one condition were discarded prior to clustering. Based on the Silhouette score, we selected *k* = 3 (score = 0.632) over *k* = 4 (score = 0.652), prioritizing simpler clustering and better biological interpretation despite the slightly lower score. Gene expression differences among clusters were addressed using Kruskal-Wallis tests followed by Dunn’s post hoc tests with Benjamini-Hochberg correction for multiple comparisons. For the GO term enrichment, we used the clusterProfiler tool v4.8.3 from Bioconductor. We ran the enrichGO function of the package for each gene cluster, using all genes from all clusters as gene universe, and searching for Biological Process GO terms. We adjusted *P* values using the Benjamini-Hochberg correction method and a *q* value cutoff of 0.2.

### METALoci analysis

METALoci (v1.3) (*43*) was used to analyze the spatial autocorrelation of epigenetic signals within the region chr2L:6,000,000–10,000,000. Epigenomic marks including H3K4me1, H3K27ac, and ATAC signals were intersected with 10-kb genomic bins using the metaloci prep command with default settings. Subsequently, the metaloci layout command generated a graph layout using the Kamada-Kawai algorithm (*42*), where each node represents a genomic bin and each edge corresponds to an interaction detected in the Hi-C contact map for the region (Fig. 4A). Parameter optimization for the layout was performed algorithmically using the *-i* flag. This process identified a cutoff threshold of 20.4% for the REG condition and 20.1% for the CTRL condition, defining the top-ranked interactions to include. In addition, a persistence length, representing the minimum distance between consecutive nodes, was set to 10.4 for REG and 10.3 for CTRL.

The LMI algorithm (*44*, *76*) was then applied using the metaloci lm command with default parameters. This step mapped the epigenetic signals onto the nodes of the layout and performed the autocorrelation analysis. For each node, the algorithm calculates an autocorrelation score based on the signal intensity of that node and those within a neighborhood defined as three times the mean distance between consecutive nodes. Each node is then classified into one of four categories based on the relationship between its signal and that of its neighborhood: high-high (red): both the node and its neighborhood exhibit high signal; low-high (cyan): the node has low signal while the neighborhood has high signal; low-low (blue): both the node and its neighborhood exhibit low signal; and high-low (orange): the node has high signal while the neighborhood has low signal. The statistical significance of autocorrelation was evaluated using a random permutation test (*n* = 9999). By applying this classification and highlighting statistically significant nodes, spatially correlated 3D hubs for H3K4me1 became visually apparent in the Gaudí plots.

To perform iterative deletions within the target region, the metaloci scan command was used, applying the same layout parameters as in the no-deletion (wild-type) layout. In this assay, a 100-kb sliding window (corresponding to 10 nodes) was sequentially removed from the node list. This operation effectively eliminated all interactions involving those nodes, as well as their associated epigenetic signals. The layout was then recalculated using the modified set of spatial restraints. For each updated layout, corresponding to the deletion of a specific anchoring node, the LMI algorithm was reapplied to evaluate the effect of the deletion on autocorrelation values, relative to the original, unaltered layout.

To calculate the correlation of predicted changes in the H3K4me1 between each anchor deletion, we first verified the assumptions of normality (using the Shapiro-Wilk test) and homoscedasticity (by examining residual plots). Then, we calculated the PCC for each pair of deleted anchors using the list of LMI differential scores calculated for each 10-kb bin. Bins with an absolute LMI differential <0.1 were discarded prior to PCC calculation to remove the effect of stable regions.

To assess the correlation between the changes in gene expression and the predicted changes in H3K4me1, we first associated each gene to its overlapping 10-kb bin. For genes overlapping >1 bins, we only selected the bin overlapping the highest proportion of the gene sequence. To take into consideration the H3K4me1 3D environment, each gene was associated with a 3D environment, defined as the sum of bins located <0.1 of distance in the Kamada-Kawai graph layout. The LMI differential for each 3D environment was then calculated as the average LMI differential of all included bins. The Spearman’s rank correlation coefficient was calculated factoring in gene expression changes (previously calculated log2FC) and the H3K4me1 LMI differential of the 3D environments. The log2FC used in each case was dependent on the deleted anchor: log2FC of *L1^Df^* was used when deleting A1 and A4, log2FC of *L2*^Δ*A3*^ was used when deleting A2 and A3, and log2FC of *L3*^Δ*A5*^ was used when deleting A2 and A5.
