# Supplementary material for: 3D genome organization in tissue regeneration involves long-range chromatin loops
Source: Sci Adv. 2026 Aug 7;12(32):eaea8281. doi: 10.1126/sciadv.aea8281 (PMC13450218; doi:10.1126/sciadv.aea8281)
Supplement: Supplementary file 1 [file sciadv.aea8281_sm.pdf]

Science Advances
AAAS

Supplementary Materials for

3D genome organization in tissue regeneration involves long-range chromatin loops

Palmira Llorens-Giralt *et al.*

Corresponding author: Marc A. Marti-Renom, martirenom@cnag.eu; Montserrat Corominas, mcorominas@ub.edu

Sci. Adv. **12**, eaea8281 (2026)
DOI: 10.1126/sciadv.aea8281

The PDF file includes:

Figs. S1 to S6
Tables S4 to S6
Legends for tables S1 to S3

Other Supplementary Material for this manuscript includes the following:

Tables S1 to S3

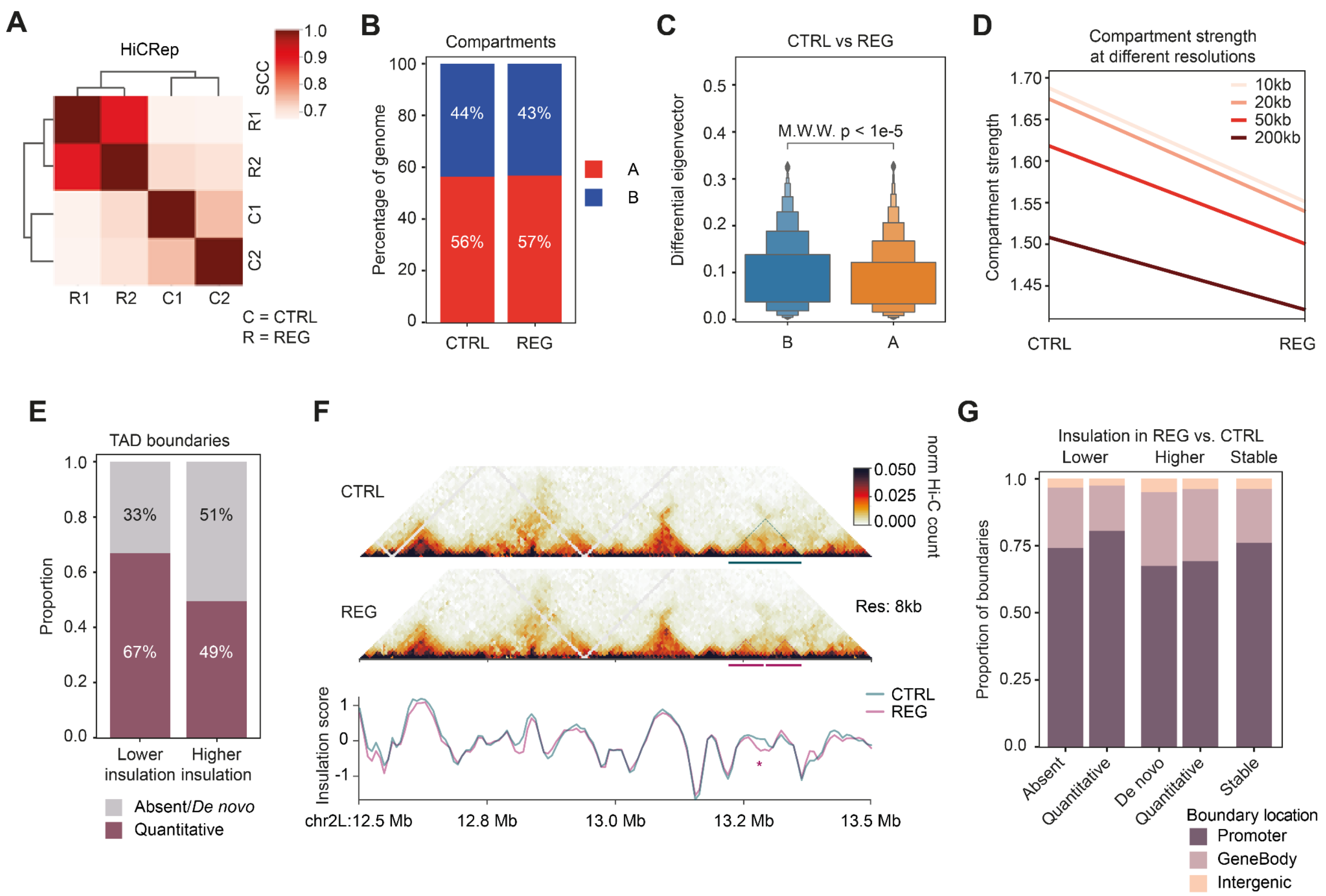

Figure S1. Genome-wide A/B compartment and boundary insulation analysis.

(A) Sample correlation analysis using HiCRep. Stratum-adjusted correlation coefficients (SCC) are shown for each pairwise Hi-C comparison. **(B)** Genome-wide proportions of A and B compartments in control and regenerating discs. **(C)** Differential eigenvector values (control vs. regeneration) for regions assigned to A or B compartments. **(D)** Compartment strength measured at multiple resolutions in control and regeneration conditions. **(E)** Proportion of topologically associating domain (TAD) boundaries classified as either absent/*de novo* or quantitative, showing lower (Lower insulation) or higher (Higher insulation) in regeneration. **(F)** Hi-C contact maps with insulation scores for CTRL and REG illustrating a *de novo* TAD boundary emerging in regeneration (asterisk). TAD length in the region of interest is shown below each Hi-C map. **(G)** Proportion of TAD boundaries located overlapping a promoter, gene body, or intergenic regions.

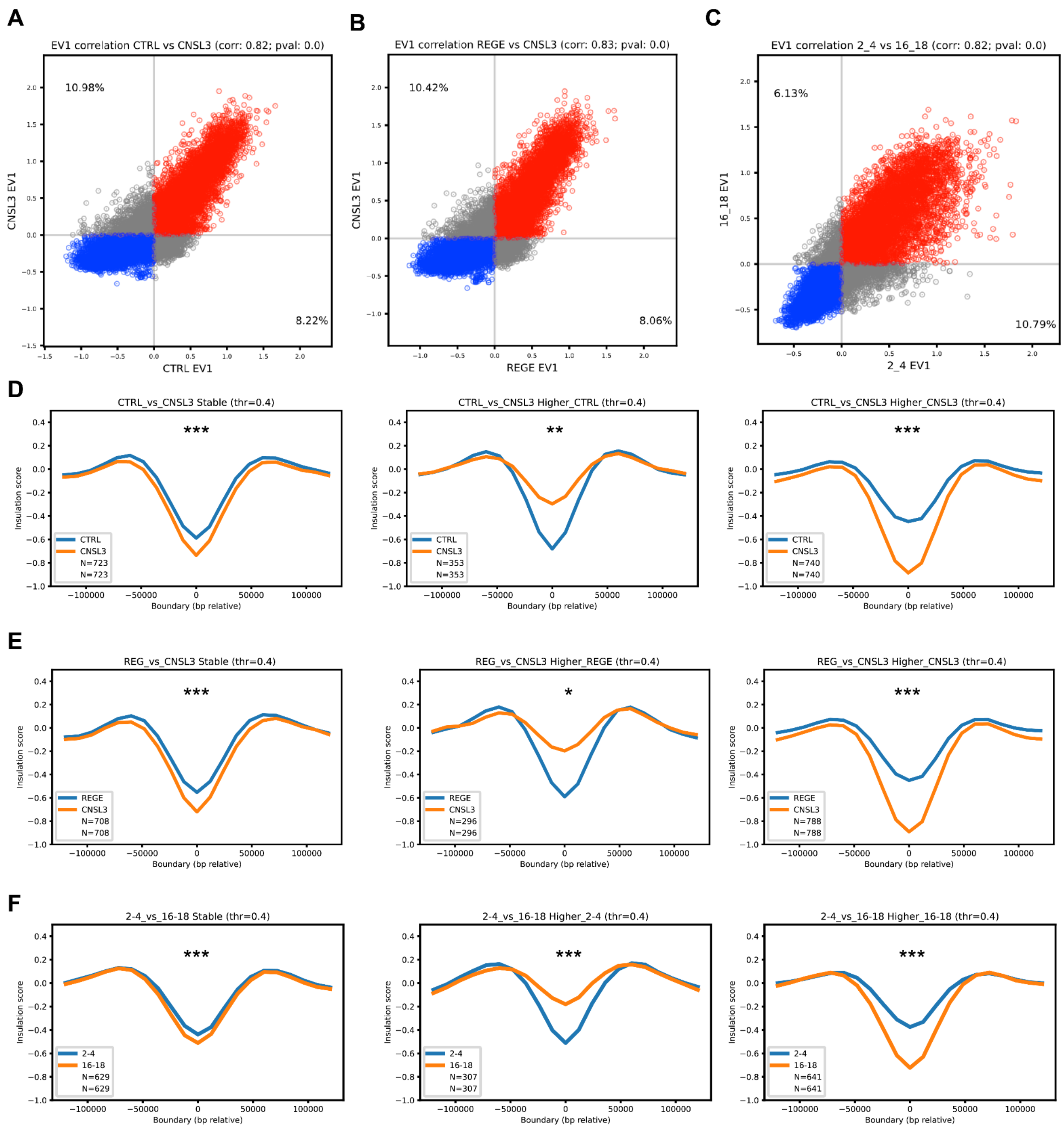

Figure S2. Compartment and insulation score comparisons to Mohana et al. (*20*) and Pollex et al. (*23*) datasets.

(A) Genome-wide comparison of Eigen values between CTRL and CNSL3 showing a 10.98% compartment change from B to A and 8.22% compartment change from A to B having CTRL as base-line. **(B)** Genome-wide comparison of Eigen values between REG and CNSL3 showing a 10.42% compartment change from B to A and 8.06% compartment change from A to B having REG as base-line. **(C)** Genome-wide comparison of Eigen Vector 1 between 6-8h and 16-18h showing a 6.13% compartment change from B to A and 10.79% compartment change from A to

B having 2-4h as base-line. **(D)** Comparison of the CTRL and CNSL3 insulation scores at TAD for “Stable”, “CTRL > CNSL3” and “CNSL3 > CTRL” boundaries on the left, middle and right, respectively. **(E)** Comparison of the REG and CNSL3 insulation scores at TAD for “Stable”, “REG > CNSL3” and “CNSL3 > REG” boundaries on the left, middle and right, respectively. **(F)** Comparison of the 2-4h and 16-18h insulation scores at TAD for “Stable”, “2-4h > 16-18h” and “16-18h > 2-4h” boundaries on the left, middle and right, respectively. Statistical significances in panels D to F are for t-test paired sample indicating ns for non-significant, * for $p<0.05$, ** for $p<0.01$, and *** for $p<0.001$.

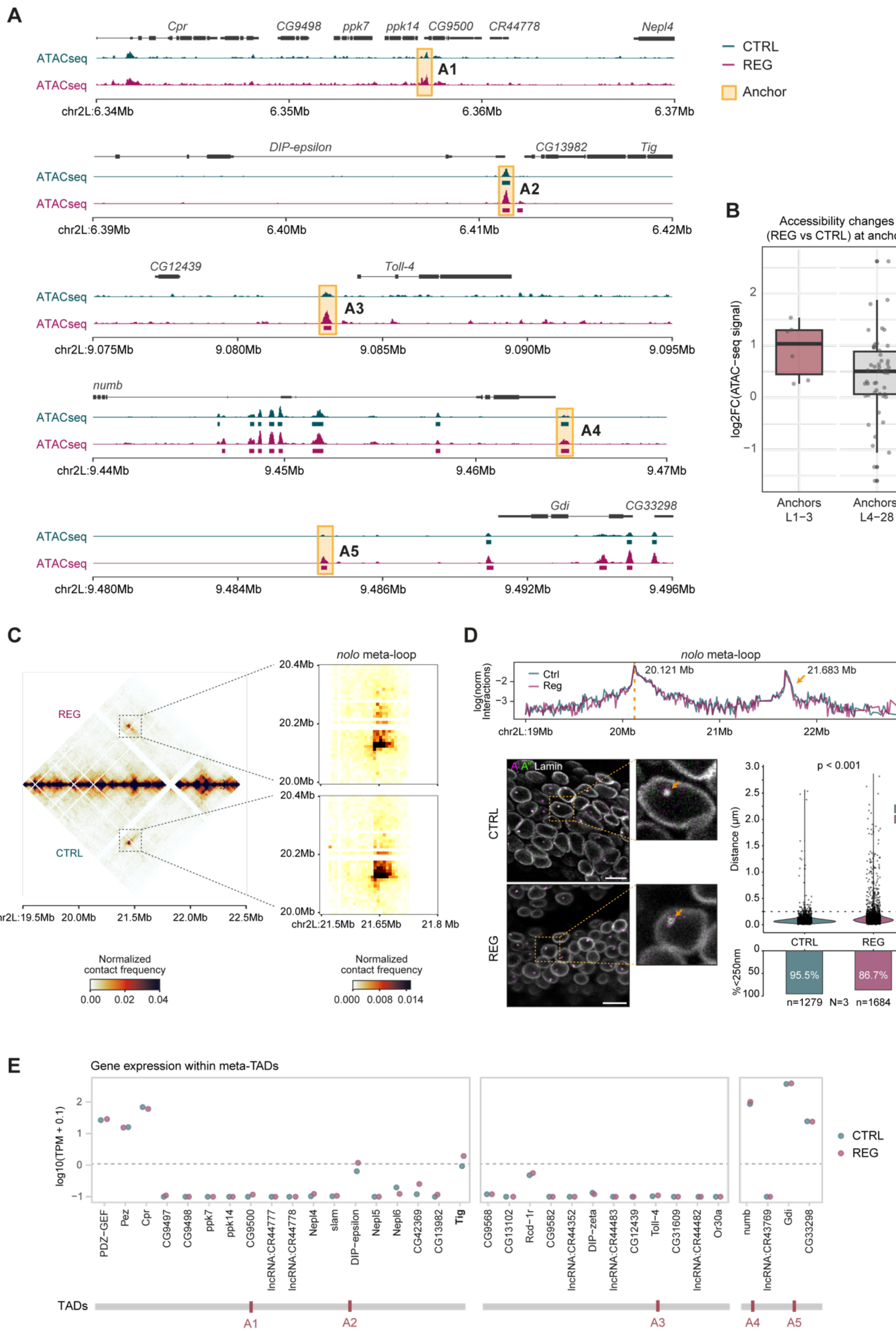
A
Cpr
CG9498
ppk7
ppk14
CG9500
CR44778
Nepl4
ATACseq
ATACseq
A1
chr2L:6.34Mb
6.35Mb
6.36Mb
6.37Mb
CTRL
REG
Anchor
DIP-epsilon
CG13982
Tig
A2
chr2L:6.39Mb
6.40Mb
6.41Mb
6.42Mb
CG12439
Toll-4
A3
chr2L:9.075Mb
9.080Mb
9.085Mb
9.090Mb
9.095Mb
numb
A4
chr2L:9.44Mb
9.45Mb
9.46Mb
9.47Mb
Gdi
CG33298
A5
chr2L:9.480Mb
9.484Mb
9.486Mb
9.492Mb
9.496Mb
B
Accessibility changes
(REG vs CTRL) at anchors
log2FC(ATAC−seq signal)
Anchors
L1−3
Anchors
L4−28
C
REG
CTRL
nolo meta-loop
20.4Mb
20.2Mb
20.0Mb
chr2L:19.5Mb
20.0Mb
21.5Mb
22.0Mb
22.5Mb
chr2L:21.5Mb
21.65Mb
21.8 Mb
Normalized
contact frequency
0.00
0.02
0.04
Normalized
contact frequency
0.000
0.008
0.014
D
nolo meta-loop
log(norm
Interactions)
Ctrl
Reg
20.121 Mb
21.683 Mb
chr2L:19Mb
20Mb
21Mb
22Mb
23Mb
A' A'' Lamin
CTRL
REG
p < 0.001
Distance (µm)
CTRL
REG
%<250nm
95.5%
86.7%
n=1279
N=3
n=1684
E
Gene expression within meta-TADs
log10(TPM + 0.1)
CTRL
REG
PDZ−GEF
Pez
Cpr
CG9497
CG9498
ppk7
ppk14
CG9500
lncRNA:CR44777
lncRNA:CR44778
Nepl4
slam
DIP−epsilon
Nepl5
Nepl6
CG42369
CG13982
Tig
CG9568
CG13102
Rcd−1r
CG9582
lncRNA:CR44352
DIP−zeta
lncRNA:CR44483
CG12439
Toll−4
CG31609
lncRNA:CR44482
Or30a
numb
lncRNA:CR43769
Gdi
CG33298
TADs
A1
A2
A3
A4
A5

Figure S3. Mapping of L1-L3 meta-loop anchors and characterization of the *nolo* meta-loop.

(A) Genome browser tracks showing gene annotations and ATAC-seq signal intensities in control and regenerating wing discs (*29*) at each meta-loop anchor region. The ATAC-seq peak assigned as the loop anchor summit is highlighted in yellow. **(B)** Boxplot showing accessibility changes for the anchors A1-A5 from meta-loops L1-L3 compared to anchors from all other identified meta-loops. Changes in chromatin accessibility were measured as the log2-transformed fold change of the ATAC-seq signal for regenerating discs compared to control discs. **(C)** Mirror plot of normalized Hi-C contact maps for regeneration (top) and control (bottom), centered on the *nolo* meta-loop region. **(D)** Top: Virtual 4C plots using Hi-C data from regeneration and control samples, with the intergenic anchor (chr2L: 20.121 Mb) as the bait to detect the loop with the *nolo* promoter (chr2L: 21.683 Mb). Bottom left: Representative DNA-FISH images of wing discs labeled with probes targeting each *nolo* loop anchor. Lamin staining (white) marks the nuclear lamina. Bottom right: Quantification of DNA-FISH distances (in µm) for the *nolo* loop is shown as violin plots. The percentage of nuclei with distances <250 nm is indicated below. *n* denotes the number of nuclei analyzed; *N* indicates the number of biological replicates. **(E)** Gene expression levels ($\log_{10}$[TPM + 0.1]) for genes located within meta-domains in control and regenerating wing discs (*37*). The dashed line indicates the expression threshold (>1 TPM). Differentially expressed genes are shown in bold. The schematic below indicates meta-domain boundaries and anchor positions.

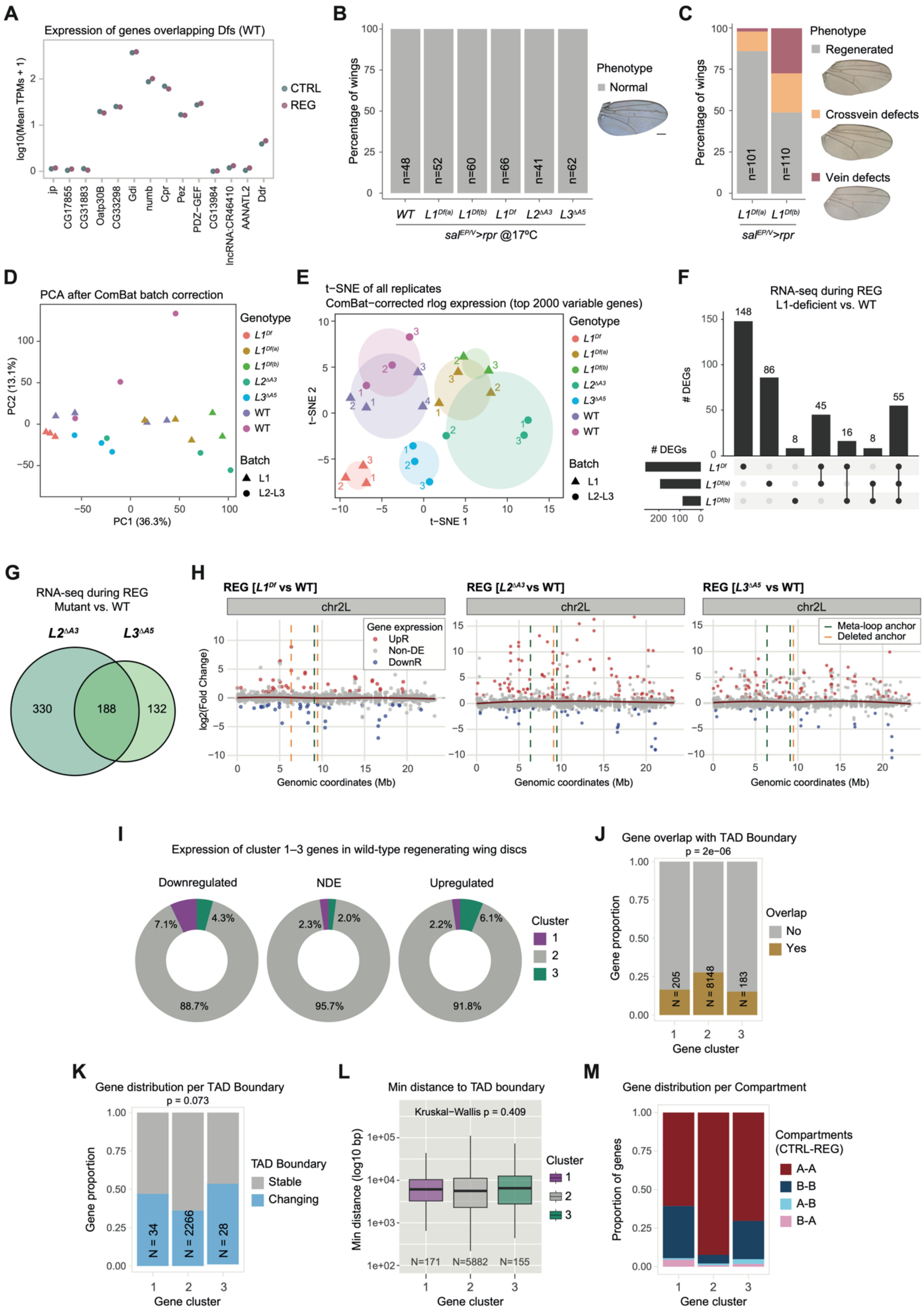

A
Expression of genes overlapping Dfs (WT)
log10(Mean TPMs + 1)
CTRL
REG
jp
CG17855
CG31883
Oatp30B
CG33298
Gdi
numb
Cpr
Pez
PDZ-GEF
CG13984
lncRNA:CR46410
AANATL2
Ddr
B
Percentage of wings
n=48
n=52
n=60
n=66
n=41
n=62
WT
L1Df(a)
L1Df(b)
L1Df
L2ΔA3
L3ΔA5
salEPV>rpr @17ºC
Phenotype
Normal
C
Percentage of wings
n=101
n=110
L1Df(a)
L1Df(b)
salEPV>rpr
Phenotype
Regenerated
Crossvein defects
Vein defects
D
PCA after ComBat batch correction
PC2 (13.1%)
PC1 (36.3%)
Genotype
L1Df
L1Df(a)
L1Df(b)
L2ΔA3
L3ΔA5
WT
WT
Batch
L1
L2-L3
E
t-SNE of all replicates
ComBat-corrected rlog expression (top 2000 variable genes)
t-SNE 2
t-SNE 1
Genotype
Batch
L1
L2-L3
F
RNA-seq during REG
L1-deficient vs. WT
DEGs
148
86
8
45
16
8
55
L1Df
L1Df(a)
L1Df(b)
G
RNA-seq during REG
Mutant vs. WT
L2ΔA3
L3ΔA5
330
188
132
H
REG [L1Df vs WT]
REG [L2ΔA3 vs WT]
REG [L3ΔA5 vs WT]
chr2L
log2(Fold Change)
Genomic coordinates (Mb)
Gene expression
UpR
Non-DE
DownR
Meta-loop anchor
Deleted anchor
I
Expression of cluster 1–3 genes in wild-type regenerating wing discs
Downregulated
NDE
Upregulated
7.1%
4.3%
88.7%
2.3%
2.0%
95.7%
2.2%
6.1%
91.8%
Cluster
1
2
3
J
Gene overlap with TAD Boundary
p = 2e−06
Gene proportion
N = 205
N = 8148
N = 183
Gene cluster
Overlap
No
Yes
K
Gene distribution per TAD Boundary
p = 0.073
Gene proportion
N = 34
N = 2266
N = 28
Gene cluster
TAD Boundary
Stable
Changing
L
Min distance to TAD boundary
Kruskal−Wallis p = 0.409
Min distance (log10 bp)
N=171
N=5882
N=155
Gene cluster
Cluster
1
2
3
M
Gene distribution per Compartment
Proportion of genes
Gene cluster
Compartments
(CTRL-REG)
A-A
B-B
A-B
B-A

Fig. S4. Transcriptomic effects of meta-loop anchor disruption during regeneration.

(A) Gene expression levels ($\log_{10}$[TPM + 0.1]) for genes overlapping deficiencies in control and regenerating wing discs (*37*). **(B)** Proportion of adult wings from mutant flies under normal conditions (17 ºC). Representative image shown to the right. **(C)** Proportion of adult wing phenotypes in $L1^{Df(a)}$ and $L1^{Df(b)}$ after cell death-induction: fully regenerated wings (grey), crossvein defects (yellow), and other vein abnormalities (rose). Representative images of each phenotype are shown to the right. **(D)** Principal Component Analysis (PCA) on the rlog-normalized gene expression values after ComBat batch correction of RNA-seq samples. **(E)** t-distributed stochastic neighbor embedding (t-SNE) plot of the top 2,000 variable genes from the same samples. Each point represents one sample in D-E, colored by experimental condition, with point shape indicating the original batch. **(F)** Upset plot of differentially expressed genes (DEGs) during regeneration in $L1^{Df(a)}$, $L1^{Df(b)}$, and $L1^{Df}$ compared to WT, showing DEG intersections across comparisons. **(G)** Venn diagram showing DEG overlap between $L2^{\Delta A3}$ and $L3^{\Delta A5}$ vs. WT flies during regeneration. **(H)** Scatter plot of gene expression changes on chromosome 2L during regeneration in each mutant compared to WT. Log2-transformed fold changes for all genes are shown as upregulated (red), downregulated (blue), or non-differentially expressed (grey), and loop anchor positions are marked as intact (green) or deleted (orange). **(I)** Distribution of upregulated, downregulated and non-differentially expressed (NDE) genes in wild-type regeneration across clusters 1-3. **(J)** Fraction of genes from each cluster overlapping TAD boundaries. **(K)** Proportion of boundary-overlapping genes associated with insulation changes during regeneration. **(L)** Minimum distance of cluster-assigned genes to the nearest TAD boundary. **(M)** Distribution of cluster-assigned genes across A/B compartments in control and regenerating discs.

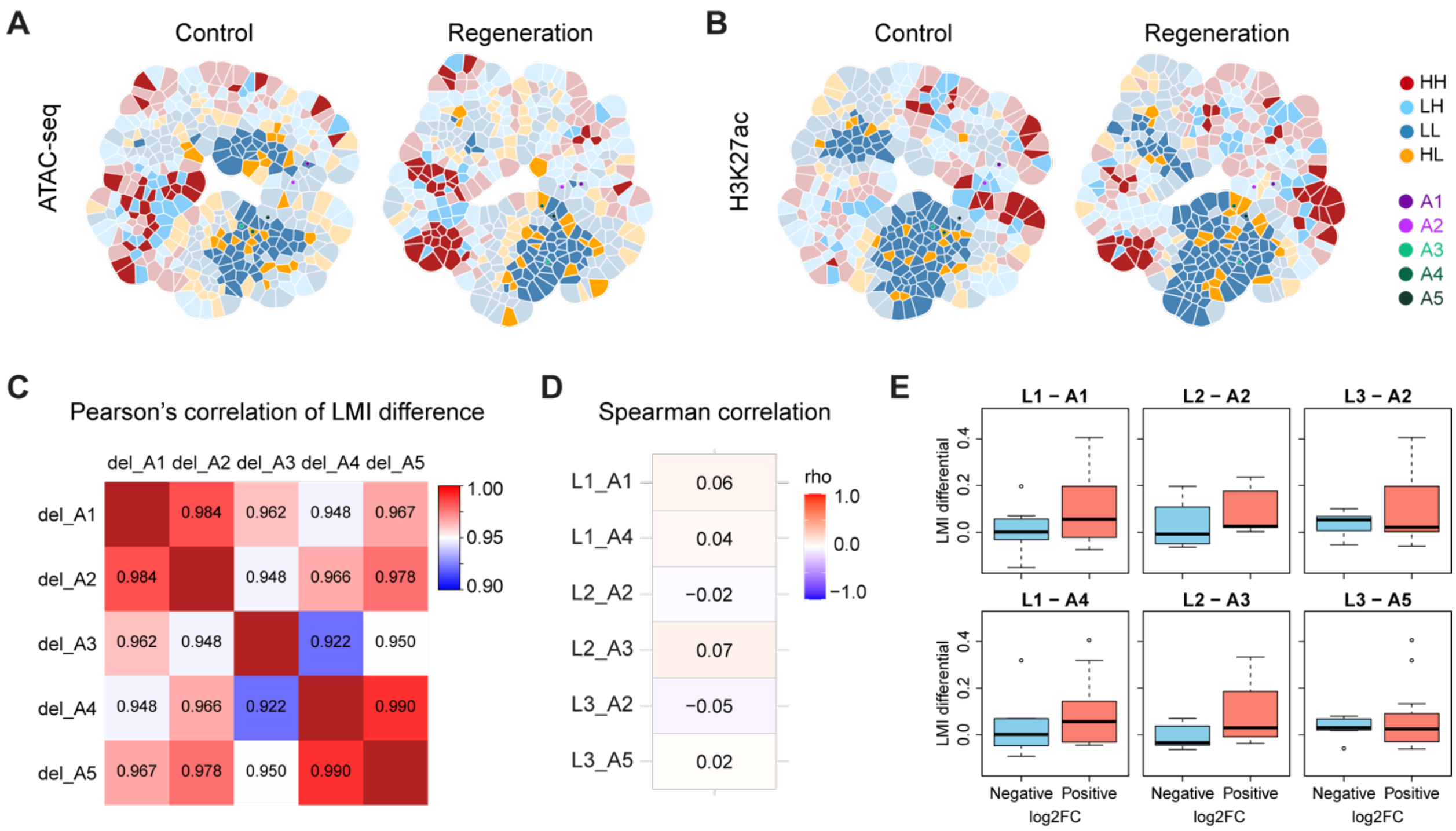

Figure S5. Overall gene expression changes do not correlate with predicted changes in the H3K4me1 landscape

(A) Gaudí plots displaying ATAC-seq signals (*27*) and spatial classification of each bin based on the Local Moran's I (LMI). Bins are color-coded to indicate quadrant classification and statistical significance (solid colors, $p < 0.05$). Bins are classified as: high–high (HH), enriched in signal and surrounded by similarly enriched bins; low–low (LL), depleted in signal and surrounded by depleted bins; high–low (HL), enriched in signal-poor neighborhoods; and low–high (LH), depleted within signal-rich neighborhoods. **(B)** Same as (A) for H3K27ac. **(C)** Pearson's correlation coefficient of the LMI differences resulting from the deletion of each meta-loop anchor region. The LMI differential for each deletion was calculated as the difference between the predicted LMI score the deletion and the score for wild type regenerating discs. **(D)** Spearman's rank correlation coefficient between log2-transformed gene expression fold changes and the 3D environment LMI differential scores. (**E**) Boxplots showing the distribution of the 3D environment LMI differential scores grouped for genes with a positive, or a negative log2FC (deletion vs. WT). Left panel plots: LMI differentials for the predicted deletions of L1 anchors A1 and A4, log2FC for the RNA-seq of $L1^{Df}$. Middle panel plots: LMI differentials for the predicted deletions of L2 anchors A2 and A3, log2FC for the RNA-seq of $L2^{\Delta A3}$. Right panel plots: LMI differentials for the predicted deletions of L3 anchors A2 and A5, log2FC for the RNA-seq of $L3^{\Delta A5}$.

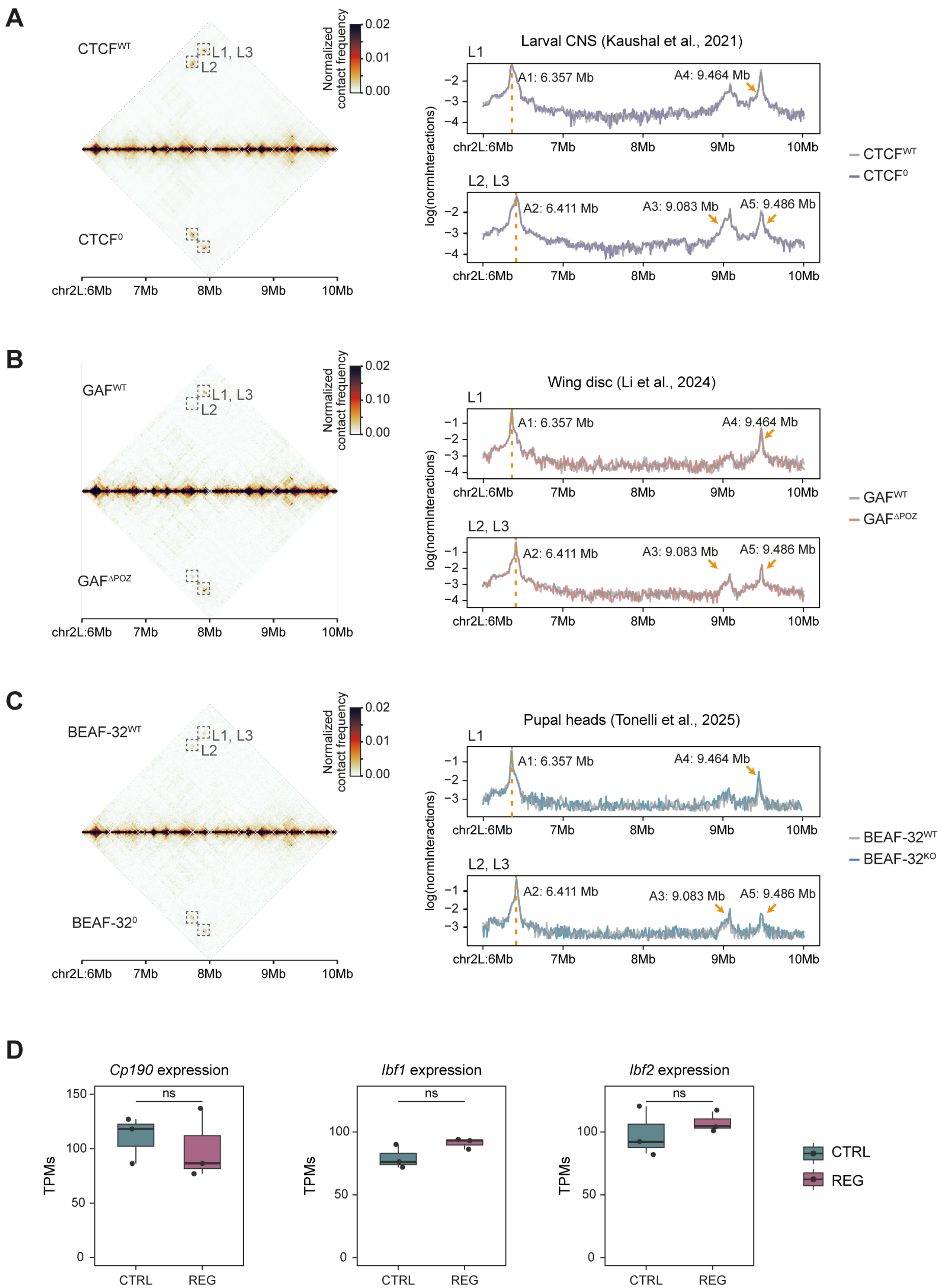

A
CTCF^WT
CTCF^0
L1, L3
L2
Normalized contact frequency
0.02
0.01
0.00
chr2L:6Mb
7Mb
8Mb
9Mb
10Mb
Larval CNS (Kaushal et al., 2021)
L1
A1: 6.357 Mb
A4: 9.464 Mb
L2, L3
A2: 6.411 Mb
A3: 9.083 Mb
A5: 9.486 Mb
log(normInteractions)
CTCF^WT
CTCF^0
B
GAF^WT
GAF^ΔPOZ
0.10
Wing disc (Li et al., 2024)
GAF^WT
GAF^ΔPOZ
C
BEAF-32^WT
BEAF-32^0
Pupal heads (Tonelli et al., 2025)
BEAF-32^WT
BEAF-32^KO
D
Cp190 expression
Ibf1 expression
Ibf2 expression
ns
TPMs
CTRL
REG

Fig. S6. CTCF and GAF are not required for meta-loop formation.

(A) Hi-C contact map analysis of larval central nervous system tissue from wild-type (top) and *CTCF*^{0} (bottom) flies (*47*). Left*:* Mirror plot of normalized Hi-C contact maps for chr2L: 6–10 Mb. Right*:* Virtual 4C plots using A1 as bait to visualize loop L1 (top), and A2 as bait to detect loops L2 and L3 (bottom). Loop anchor coordinates are indicated. **(B)** Same as (A) but comparing wild-type (top) and *GAF* mutant (bottom) wing discs (*46*). **(C)** Same as (A) but comparing wild- type (top) and BEAF-32-deficient (maternal depletion and knockout, *BEAF-320*) (bottom) pupal heads (*59*). **(D)** Expression levels (TPM) of *Cp190*, *Ibf1*, and *Ibf2* in control and regenerating wing discs (*37*).

Table S4. Primers used for CRISPR-Cas9-mediated anchor deletions

Primer name	Primer sequence (5'>3')	Purpose
A3_gRNA 1 FW	GTCGATAAGAAGTAGTCCTTGCAT	sgRNA for CRISPR KO
A3_gRNA 1 RV	AAACATGCAAGGACTACTTCTTAT	sgRNA for CRISPR KO
A3_gRNA 2 FW	TTCGTTTGAGCGGGACTCAGCGGC	sgRNA for CRISPR KO
A3_gRNA 2 RV	AAACGCCGCTGAGTCCCGCTCAAA	sgRNA for CRISPR KO
A3_gRNA 3 FW	CTTCGGTTCTTATAATAATTGCATT	sgRNA for CRISPR KO
A3_gRNA 3 RV	AAACAATGCAATTATTATAAGAACC	sgRNA for CRISPR KO
A3_ gRNA 4 FW	GTCGATATATAGTAACTGCAACAA	sgRNA for CRISPR KO
A3_ gRNA 4 RV	AAACTTGTTGCAGTTACTATATAT	sgRNA for CRISPR KO
A5_gRNA 1 FW	GTCGTAGTATAAGCTATGATTATC	sgRNA for CRISPR KO
A5_gRNA 1 RV	AAACGATAATCATAGCTTATACTA	sgRNA for CRISPR KO
A5_ gRNA 2 FW	TTCGATATATCGTATTATCTCATT	sgRNA for CRISPR KO
A5_ gRNA 2 RV	AAACAATGAGATAATACGATATAT	sgRNA for CRISPR KO
A5_gRNA 3 FW	CTTCGAAGTTCTGTATATTTAGAGA	sgRNA for CRISPR KO
A5_gRNA 3 RV	AAACTCTCTAAATATACAGAACTTC	sgRNA for CRISPR KO
A5_gRNA 4 FW	GTCGGCTGAAAAGATACAAGCTAA	sgRNA for CRISPR KO
A5_gRNA 4 RV	AAACTTAGCTTGTATCTTTTCAGC	sgRNA for CRISPR KO

Table S5. Hi-C mapping results

ID	Reads	Intersect	%	Valid interactions	%	CIS%	Dangling	%
**CTRL_1**	570,869,344	559,409,126	98.0%	295,967,093	51.8%	72.0%	33,664,060	6%
**CTRL_2**	580,056,454	570,516,966	98.4%	313,422,371	54.0%	73.0%	33,846,233	6%
**REG_1**	461,920,910	453,990,777	98.3%	273,564,949	59.2%	69.0%	23,382,502	5%
**REG_2**	563,556,925	550,153,826	98.6%	331,416,207	58.8%	68.0%	28,693,391	5%

ID	Extra DE	%	Duplicates	%	R. Breaks	%	Error	%	Self circle	%
**CTRL_1**	100,437,826	18%	146,411,350	26%	1,940,749	0%	45,146,122	8%	395,628	0%
**CTRL_2**	100,437,826	17%	134,975,570	23%	1,698,931	0%	43,223,663	7%	421,745	0%
**REG_1**	73,685,374	16%	77,345,636	17%	1,001,857	0%	27,256,413	6%	296,082	0%
**REG_2**	89,468,603	16%	96,649,021	17%	1,231,786	0%	33,214,971	6%	336,868	0%

Table S6. NCBI GEO Accession numbers

GEO accession	Experiment	Type of Data	doi
GSE304452	Hi-C	Wing discs (CTRL, REG)	Present study
GSE304453	RNA-seq	Wing discs REG (WT, *L1*$^{Df(a)}$, *L1*$^{Df(b)}$, *L1*^{Df}, *L2*$^{\Delta A3}$ and *L3*$^{\Delta A5}$)	Present study
GSE223411	RNA-seq	Wing discs (CTRL, REG)	10.1093/nargab/lqae091
GSE102841	ATAC-seq	Wing discs (CTRL, REG)	10.1101/gr.233098.117
GSE102841	ChIP-seq	H3K27ac and H3K4me1 in wing discs (CTRL, REG)	10.1101/gr.233098.117
GSE180376	ChIP-seq	Cp190 and CTCF in embryos	10.1126/sciadv.abl8834
GSE218168	ChIP-seq	GAF in wing discs	10.1016/j.molcel.2023.03.011
GSE47559	ChIP-seq	Ibf1 and Ibf2 in S2	10.1002/embj.201386001
GSE80700	ChIP-seq	Pita in Kc167	10.1093/nar/gkw1114
GSE30740	ChIP-seq	Su(Hw) and BEAF-32 in Kc167	10.1038/ncomms2469
GSE49842	ChIP-seq	M1BP in S2 cells	10.1038/emboj.2013.111
GSE54337	ChIP-seq	ZIPIC in S2 cells	10.1101/gr.174169.114
GSE286361	Hi-C	Embryonic neurons (WT, Cp190[0])	10.1101/gad.352646.125
GSE146752	Hi-C	Larval CNS (WT, CTCF[K0])	10.1038/s41467-021-21366-2
GSE218168	Hi-C	Larval CNS (WT, GAF[POZ])	10.1016/j.molcel.2023.03.011
GSE253140	Hi-C	Pupal heads (WT, BEAF-32[0])	10.1016/j.devcel.2024.10.017

Table S1. (separate file)
Meta-loops identified in the wing discs using the loop calling algorithm from Mohana *et al*. (*20*). Strength values (observed contacts) for control and regeneration, as well as the log2-transformed fold changes and associated *p*-values are shown.

Table S2. (separate file)
Summary of *de novo* motifs identified at chromatin loop anchors. For each motif, the table reports: motif sequence, STREME enrichment score, best match in the JASPAR CORE Insect database, associated statistics (*p*-value, *E*-value, *q*-value), query and target consensus sequences, distribution across anchor groups, adjusted *p*-values from Fisher's exact test, annotation status, and mean TPM expression values in control and regeneration conditions in the wing disc.

Table S3. (separate file)
Gene list showing the *k*-means cluster ID, as well as the log2-transformed fold changes and adjusted *p*-values of regenerating wing discs from the 3 transcriptomes of loop-deficient flies ($L1^{Df}$, $L2^{\Delta A3}$, and $L3^{\Delta A5}$).
